# PLXNC1 Enhances Carcinogenesis Through Transcriptional Activation of IL6ST in Gastric Cancer

**DOI:** 10.3389/fonc.2020.00033

**Published:** 2020-02-04

**Authors:** Jie Chen, Haining Liu, Jinggui Chen, Bo Sun, Jianghong Wu, Chunyan Du

**Affiliations:** ^1^Department of Gastric Surgery, Fudan University Shanghai Cancer Center, Fudan University, Shanghai, China; ^2^Department of Gastroenterology and Hepatology, Zhongshan Hospital, Fudan University, Shanghai, China

**Keywords:** transcriptional factor, PLXNC1, IL6ST, gastric cancer, carcinogenesis

## Abstract

**Background:** Transcriptional factors (TFs) are responsible for orchestrating gene transcription during cancer progression. However, their roles in gastric cancer (GC) remain unclear.

**Methods:** We analyzed the differential expressions of TFs and, using GC cells and tissues, investigated plexin C1 (PLXNC1) RNA levels, as well as PLXNC1's clinical relevance and functional mechanisms. The molecular function of PLXNC1 was evaluated *in vitro* and *in vivo*. Kaplan-Meier curves and the log-rank test were used to analyze overall survival (OS) and disease-free survival (DFS).

**Results:** PLXNC1 was frequently up-regulated in GC and associated with poor prognosis. The expression level of PLXNC1 could serve as an independent biomarker to predict a patient's overall survival. Notably, knockdown of PLXNC1 significantly abolished GC cell proliferation, and migration, and overexpression of PLXNC1 accelerated carcinogenesis in GC. The gene set enrichment analysis (GSEA) indicated that high-expression of PLXNC1 was positively correlated with the activation of epithelial-mesenchymal transition (EMT), TNF-α, and IL-6/STAT3 signaling pathways. PLXNC1 promoted proliferation and migration of GC cells through transcriptional activation of the interleukin 6 signal transducer (IL6ST), which could rescue the malignant behavior of PLXNC1-deficient GC cells.

**Conclusions:** Our study demonstrated that the PLXNC1 plays an oncogenic role in GC patients. The PLXNC1-IL6ST axis represents a novel potential therapeutic target for GC.

## Introduction

Gastric cancer (GC) is one of the most malignant and prevalent tumors, with poor prognosis worldwide (1, 2). Although clinical therapeutic methods and medical technology have improved (surgical resection and target drug therapy, for example), the 5 year survival rates of GC still remain dismal (3). Moreover, the molecular mechanism underlying gastrocarcinogenesis has not yet been completely elucidated. However, recently, genomic technology has become the essential methodology used by international organizations to discover the novel therapeutic targets in GC (4, 5). The Cancer Genome Atlas (TCGA) has carried out a systematic and multidimensional repertoire of genomic dysregulations, including gene expression, gene-level-mutation, copy number variation, and clinical information for stomach adenocarcinoma (STAD). The open-source TCGA dataset provides a suitable repository for investigators to explore new methods for GC diagnosis, treatment, and prevention (6).

Transcriptional dysregulation is a hallmark of cancer (7). Transcription factors (TFs), chromatin regulators, and other co-factors jointly regulate this process. Master, signaling, and proliferation are the major classes of TFs, and could remodel chromatin status and manipulate the generation of addictive cancer transcripts (8). In GC, kruppel-like factor 5 (KLF5) and MYC proto-oncogene bHLH transcription factor (MYC) collectively regulate long intergenic non-protein coding RNA 346 (LINC00346), thus contributing to GC progression (9). Nevertheless, the roles of TFs and their regulated targets in GC remain elusive.

In this study, we examined the latest TF catalog, comprising 1,935 TF genes (10), and systematically analyzed their transcription profile in TCGA-STAD cohort to assess the roles of TFs in GC. We identified 419 up-regulated and 64 down-regulated TF genes in STAD paired tissues. Among the TFs identified, 189 targets showed a positive correlation with patient prognosis. Moreover, we found a transcriptional factor plexin C1 (PLXNC1), which was significantly up-regulated and correlated with poor outcomes in GC patients. Notably, the PLXNC1 promoted GC cell proliferation and metastasis by enhancing tumor-related signaling pathways and transcriptional activation of IL6ST. Our results demonstrated that the PLXNC1-IL6ST axis could be a promising therapeutic target in GC.

## Materials and Methods

### Human Tissues and Follow-Up

Gastric cancer specimens and matched adjacent non-tumor tissues (NTs) from 111 patients were obtained from the Department of Gastric Surgery, Fudan University Shanghai Cancer Center, Fudan University (Shanghai, China) to analyze PLXNC1 mRNA levels. Upon resection, the tissue samples were snap-frozen in liquid nitrogen and stored at −80°C. Informed consent was acquired from all patients. The study was approved by the Ethics Committee of Shanghai Medical College of Fudan University.

### Statistical Analysis

For comparisons of two groups, statistical significance for normally distributed variables were estimated using unpaired Students *t*-test, and non-normally distributed variables were analyzed by Mann-Whitney *U*-test (also called the Wilcoxon rank-sum test). The differentially expressed genes were analyzed from moderate students t-test using the *limma* package. The Kaplan-Meier method was used to generate survival curves for the two subgroups of the binomial variables, and the Log-rank (Mantel-Cox) test was used to determine the statistical significance of the differences between survival curves. The hazard ratios for uni- and multivariate analyses were calculated by the uni- and multivariate Cox proportional hazards regression model.

The diagnostic efficiency of PLXNC1 and CEA for patients' OS times was estimated using receiver operating characteristic (ROC) curves. From a comparison of two ROC curves and the areas under the curves (AUC), 95% confidence intervals were calculated, according to the DeLong method. All statistical analyses were carried out using the R language (version 3.5.2, https://www.r-project.org/). The statistical tests were two-sided, and a *P* < 0.05 was considered statistically significant. The following R packages were used in this study: “pROC,” “rms,” “survival,” “clusterProfiler,” and “pheatmap.”

### Cell Lines and Cell Culture

The human GC cell lines (HGC-27 and AGS) were purchased from the American Type Culture Collection (ATCC) (Manassas, VA, USA). The human embryonic kidney 293T (HEK-293T) cells were purchased from the Shanghai Cell Bank Type Culture Collection Committee (CBTCCC) (Shanghai, China). HGC-27 and AGS cells were cultured in RPMI1640 (Thermo Fisher Scientific, Waltham, MA, USA) and HEK-293T cells in DMEM (Gibco, Grand Island, NY, USA), supplemented with 10% fetal bovine serum (Gibco), 100 μg/ml penicillin (Gibco), and 100 μg/ml streptomycin (Gibco), at 37°C and 5% CO_2_. Cells were treated with Mycoplasma-OUT (Genechem, Shanghai, China) for 1 week before a routine experiment and mycoplasma testing was performed by PCR.

### RNA Extraction, Reverse Transcription, and qRT-PCR Analysis

Total RNA was extracted from GC or non-tumor tissues or cells using the TRIzol reagent (Invitrogen, Carlsbad, CA, USA). cDNA was synthesized using the PrimeScript RT Reagent Kit (TaKaRa, Shiga, Japan). The quantitative real-time polymerase chain reaction (qPCR) analyses were performed using SYBR Premix *Ex Taq II* assays (TaKaRa), determined using the QuantStudio 7 Flex sequence detection system (Thermo Fisher Scientific), and calculated and normalized to β-actin using the comparative CT method [2^−Δ*CT*(*targetgene*−β−*actin*)^]. The sequences of the target gene primers used are listed in Table S1; β-actin was used as an internal control.

### RNA Interference

Small interfering RNA (siRNA) oligonucleotides targeting PLXNC1 were designed and synthesized by RiboBio (Guangzhou, China). Cells were transfected with siRNAs using the Lipofectamine RNAiMAX reagent (Invitrogen) at a final concentration of 50 nM. Cells were used for RNA extraction, proliferation, migration, and immunoblotting assays after transfection for 48 h. The sequences for the PLXNC1 siRNAs used are listed in Table S1.

### Lentivirus Production and Transduction

The packaging plasmid psPAX2 and the VSV-G envelope plasmid pMD2.G (gifts from Dr. Didier Trono), coupled with PLXNC1, Cas9, GFP overexpression plasmids, or PLXNC1 sgRNAs plasmids, were transfected into HEK293T cells using Lipofectamine 2000 (Invitrogen). Lentiviral particles were harvested at 48 h after transfection, and GC cells were infected with recombinant lentivirus plus 8 μg/mL polybrene (Sigma-Aldrich, St. Louis, MO, USA).

### Colony Formation and Migration Assays

For the colony formation assay, 1.5 × 10^3^ cells were seeded in a 6-well plate per well and incubated at 37°C for nearly 10 days. The number of colonies stained with 100% methanol containing 0.5% crystal violet (Sigma-Aldrich) was counted and analyzed. For cell migration assays, a total of 5 × 10^4^ cells were suspended per well in the upper chamber (BD Biosciences, Franklin Lakes, NJ) with 200 μL of RPMI1640 [minus fetal bovine serum (FBS)] in a 24-well plate; 800 μL of RPMI1640, supplemented with 10% FBS, was added to the lower chamber. After 20 h of incubation, the chambers were fixed and stained with 100% methanol containing 0.5% crystal violet (Sigma-Aldrich) for 20 min, followed by imaging and counting under an inverted microscope (Olympus, Tokyo, Japan).

### Xenograft in Nude Mice

PLXNC1 knockdown AGS cells and control cells were harvested and suspended in RPMI1640 without FBS. A total of 12 mice (male BALB/c-nu/nu, 6 weeks old) were randomly divided into two groups and subcutaneously injected in the lower back with 2 × 10^6^ cells in 200 μL of RPMI1640 without FBS. The mice were sacrificed, and the tumors were dissected and weighed ~5 weeks after injection. The mouse experiments were conducted using the Guide for the Care and Use of Laboratory Animals of Fudan University and approved by the Committee on the Ethics and Welfare of Laboratory Animal Science of Fudan University.

### Chromatin Immunoprecipitation-Quantitative PCR (ChIP-qPCR)

AGS cells were cross-linked for about 10 min in 1% formaldehyde, quenched in glycine, re-suspended in ChIP lysis buffer (20 mM Tris-HCl pH 7.5, 150 mM NaCl, 1% NP-40, 0.02% SDS, 5 mM EDTA, proteinase inhibitor), sonicated, and centrifuged. The supernatant was collected and incubated with Flag antibody and Dynabeads® Protein G (Thermo Fisher Scientific). The beads complex was washed five times with ChIP lysis buffer, decrosslinked and digested with RNase A and proteinase K. DNA samples were collected using MinElute Reaction Cleanup Kit (Qiagen, Hilden, Germany). ChIP-qPCR was performed using the QuantStudio 7 Flex sequence detection system (Thermo Fisher Scientific). Primers are listed in Table S1.

### Dual-Luciferase Assay

The dual-luciferase assay was conducted using the Dual-Luciferase Reporter Assay (promega). Briefly, AGS cells were transfected with luciferase, renilla, and PLXNC1-mixed siRNAs or negative control-siRNA. Cells were lysed, added with luciferase and renilla substrate, then measured after 24 h.

### Western Blotting

Proteins were separated on 10% SDS-PAGE and transferred to a nitrocellulose membrane (Bio-Rad, Hercules, CA, USA). The membrane was blocked with 5% non-fat milk and incubated with primary antibodies, followed by horseradish peroxidase-conjugated secondary antibodies. The protein bands were visualized using enhanced chemiluminescence reagents (Thermo Fisher Scientific) and Tanon 5200 Chemiluminescent Imaging System (Tanon, Shanghai, China) detection. The antibodies used are offered in Table S2.

## Results

### Transcription Factors Are Differentially Expressed With Clinical Significance in GC

**Figure 1:**
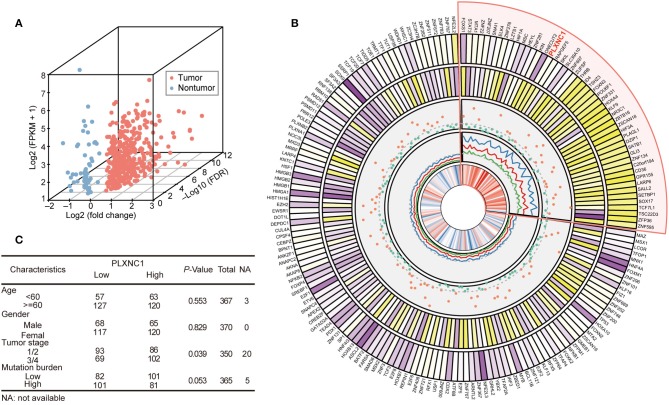
Differentially expressed transcription factors predict a patient's overall survival in TCGA-STAD cohort. **(A)** Three-dimensional scatter plot generated from the differential expression profiles of TCGA-STAD 27 paired gastric tissue. **(B)** The circled diagram of differentially expressed TF genes in TCGA-STAD cohort. In the figure comprised of five tracks, the first track refers to the average expression level (log2 transfer) of TFs; the second track indicates the fold change of differential expression analysis of TFs in the STAD paired tissue dataset; the third track shows the *P*-value (–log10 transfer) of log-rank test for each TF; the fourth track represents the hazard ratio value of univariate-cox model (HR value and it's 95% CI (lower and upper) were both highlighted as red, blue, and green ligatures, respectively); the last track displays the correlation coefficients between tumor stage and TF expression level. The darker color indicates a higher quantitative value to distinguish TFs. The sector with light red shows the high-risk TFs, which indicate poorer outcomes for GC patients. **(C)** Correlation of clinicopathological features with tumor PLXNC1 expression level in TCGA-STAD cohort.

We analyzed the expression profile of 1,935 TFs in TCGA-STAD cohort (370 samples) to explore the dysregulated levels and potential clinical significance of TFs in GC development. Twenty-seven paired tissue samples (tumor and adjacent tissues) were used to perform differential expression analysis. The results showed that 372 TFs were highly expressed in GC compared with para-cancerous samples, whereas 63 TFs were down-regulated in tumor tissues (FDR < 0.05, fold change > 1.3; Figure 1A; Table S3).

Analysis was first carried out to determine the correlation between these dysregulated TFs and OS, and to investigate the prognostic significance of TFs in GC. The clinical characteristics and whole TF expression profile (FPKM normalization) of 370 tumor samples were acquired for survival analyses. The samples were classified into two groups according to their optimal survival cut-off point for each TF, and the difference of accumulated survival curve was represented by Kaplan-Meier analysis (see Methods). The prognostic risk estimation of TFs was performed by the univariate cox proportional hazard model. Consequently, 29 down-regulated and 150 up-regulated TFs were significantly correlated with patient OS (*P* < 0.05; Figure 1B). Among them, 49 TFs showed a high risk for patient prognosis (hazard ratio > 1; highlighted in light red). Moreover, we completely analyzed the candidate-dysregulated TFs and their expression levels, hazard ratio, and correlation with tumor stages in TCGA-STAD cohort. Additionally, we investigated a possible correlation between clinical characteristics and PLXNC1 expression levels in TCGA -STAD patients, finding that GC patients with high PLXNC1 mRNA expression levels had a significant correlation with the tumor stage (Figure 1C). These results indicated that a group of TFs was dysregulated in GC, including PLXNC1, strongly correlating with clinical significance.

### High Expression of PLXNC1 Predicts Poor Prognosis in GC

**Figure 2:**
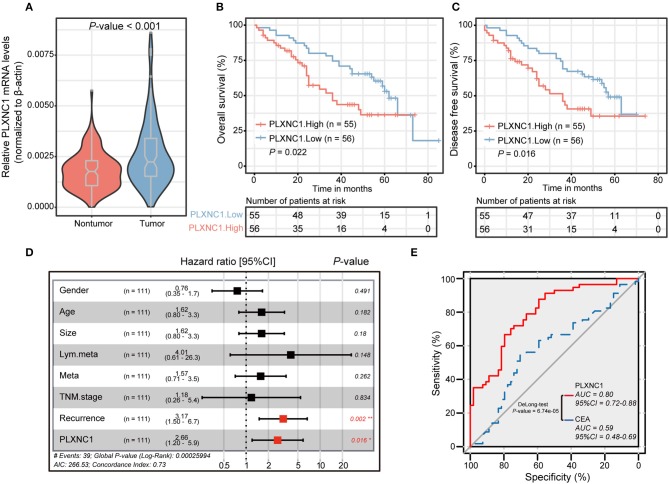
PLXNC1 predicts prognosis in gastric cancer. **(A)** The differential expression level of PLXNC1 expressed in our 111 paired STAD tissues. **(B,C)** Kaplan–Meier curves of overall survival and disease-free survival in our internal 111 gastric patients, validated by PLXNC1 mRNA expression levels. **(D)** The results of multi-variate analyses using the Cox proportional hazard regression model for PLXNC1 mRNA levels and other clinical indices in our internal cohort. **(E)** The comparison of diagnostic efficacy of CEA and PLXNC1 mRNA levels for predicting the time period of tumor OS. *P < 0.05; **P < 0.01.

We carried out quantitative real-time polymerase chain reaction (qRT-PCR) on our internal GC cohort (*n* = 111) to reveal the differential expressions of PLXNC1 in GC tissues and paired non-tumorous tissues (NTs). Importantly, the PLXNC1 was significantly up-regulated in GC samples compared with NTs at mRNA level (*P* < 0.001; Figure 2A). Kaplan-Meier Survival analysis showed that GC patients with high PLXNC1 expression levels exhibited poor OS and disease-free survival (DFS) (*P* < 0.05; Figures 2B,C). We applied multivariate analyses using the Cox proportional hazard regression model, comparing PLXNC1 expression values with other clinical factors (e.g., age, gender, tumor size, tumor stage, number of lymph node metastasis, recurrence status) as covariates, to investigate whether the expression levels of PLXNC1 were an independent prognostic factor in our internal GC cohort (*n* = 111). GC patients with a high expression level of PLXNC1 in tumors harbored a 2.66-fold high risk of death (*P* < 0.05, 95% CI, 1.20–5.90; Figure 2D).

We then investigated the effects of PLXNC1 on survival prediction by comparing it with the GC traditional diagnostic biomarker, carcinoembryonic antigen (CEA). For biopsy-proven GC patients, the expression levels of PLXNC1 and serum CEA levels (ng/ml) were used to construct a ROC curve which could evaluate the diagnostic efficiency of GC patient survival in our cohort. Consequently, PLXNC1 exhibited higher diagnostic efficacy than CEA for prediction of patient survival time (*P* < 0.001; Figure 2E). These results therefore showed that the PLXNC1 could serve as a promising prognostic biomarker for GC patients.

### PLXNC1 Plays Oncogenic Roles in GC

**Figure 3:**
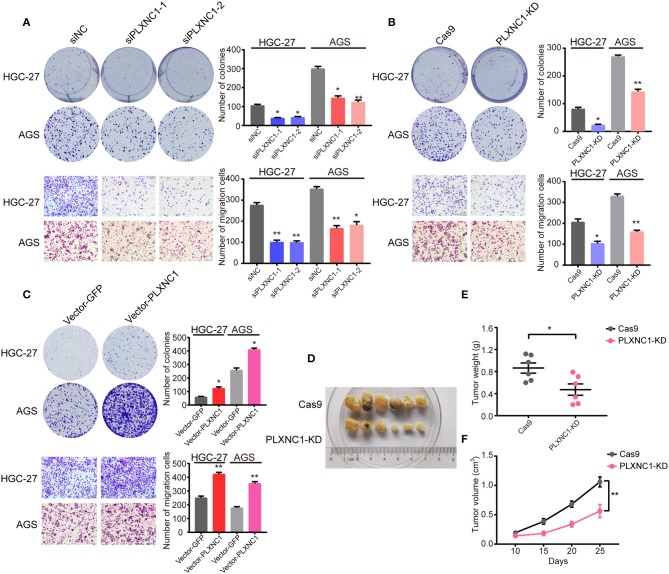
PLXNC1 plays oncogenic roles in gastric cancer both *in vitro* and *in vivo*. **(A)** Colony formation assays (up) and Transwell migration assays (down) for HGC-27 and AGS cells transfected with PLXNC1 siRNAs or negative control (NC) siRNA. **(B)** Colony formation assays (up) and Transwell migration assays (down) for HGC-27 and AGS cells infected with the PLXNC1 knockdown-mixed sgRNAs or control sgRNA lentivirus. **(C)** Colony formation assays (up) and Transwell migration assays (down) for HGC-27 and AGS cells infected with PLXNC1 overexpression lentivirus or GFP control. **(D)** Xenograft tumors of Cas9 or PLXNC1 knockdown AGS cells in nude mice. **(E,F)** The knockdown of PLXNC1 reduces the **(E)** weight and **(F)** volume of xenograft tumors (*n* = 6 mice per group). Values represent the mean ± SEM, **(A–C)** *n* = 3 and **(D–F)** *n* = 6. *P < 0.05; **P < 0.01.

We first designed two independent siRNAs targeting PLXNC1, in order to elucidate the molecular function of PLXNC1 in GC. Western blot analysis identified efficient siRNA-mediated knockdown of PLXNC1 in both HGC-27 and AGS gastric cell lines (Figure S1A). Knockdown of PLXNC1 significantly diminished GC cell proliferation and migration, as determined by colony formation and cell migration assays compared to cells treated with control siRNA (siNC) (Figure 3A). We then used lenti-clustered regularly interspaced short palindromic repeats (CRISPR) deletion systems to knockdown PLXNC1 (Figure S1B). Consistently, PLXNC1 knockdown in HGC-27 and AGS cells markedly abolished proliferation and migration (Figure 3B). We also constructed PLXNC1 overexpression lentivirus and found that overexpression of PLXNC1 in HGC-27 and AGS cells (Figure S1C) enhanced gastric cell proliferation and migration (Figure 3C). AGS cells infected with the PLXNC1 knockdown lentivirus and the control lentivirus were subcutaneously injected into the flanks of 6-week-old nude mice, then monitored for tumor growth for 5 weeks to further explore the effect of PLXNC1 on tumorigenicity. Importantly, knockdown of PLXNC1 protein expression decreased tumorigenicity (Figure 3D), as measured by the tumor weight (Figure 3E) and size (Figure 3F). In summary, these data suggest that PLXNC1 promoted carcinogenesis of GC both *in vitro* and *in vivo*.

### PLXNC1 Activates Cancer-Associated Signatures in GC

**Figure 4:**
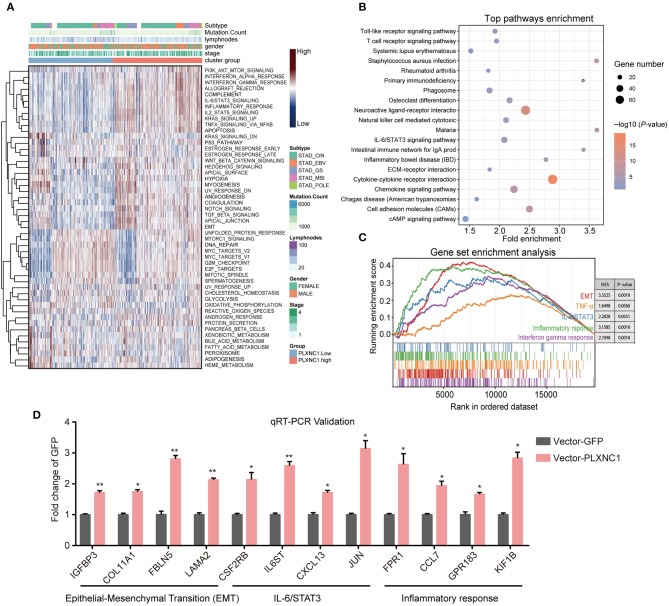
PLXNC1 activates cancer-associated signatures in gastric cancer. **(A)** The heatmap of hierarchical clustering shows the enrichment score of cancer hallmark gene sets enriched in the PLXNC1 high/low expressed group based on single sample gene set enrichment analysis (GSEA) for TCGA-STAD cohort. **(B)** KEGG pathway analysis performed by the DAVID platform for PLXNC1 high-expressed group. The KEGG pathway with *P* < 0.05 is shown in a bubble plot. **(C)** GSEA of hallmark gene sets in high-level-group of PLXNC1. All transcripts were ranked by log2 (fold change) between two groups. Each run was performed with 500 permutations. Enrichment results with significant associations with PLXNC1 were shown. **(D)** The relative candidate cancer hallmark pathway gene mRNA expression infected with PLXNC1 or GFP overexpression lentivirus in AGS gastric cancer cells using qRT-PCR analysis. **(D)** Values represent the mean ± SEM, *n* = 3. *P < 0.05; **P < 0.01.

We further explored the potential downstream targets and cancer-related signaling pathways controlled by PLXNC1. We first separated TCGA-STAD samples into two groups (high and low PLXNC1-expression level sub-groups) according to the PLXNC1 median value. Next, we performed the single-sample gene set enrichment method (ssGSEA) to evaluate the enrichment degree of 50 cancer hallmark gene signatures in whole 370 GC samples. Gene set enrichment scores for each sample were further clustered by hierarchical agglomerative clustering (Ward's linkage). The results demonstrated that the TNF-α, IL-6/STAT3 pathway, inflammatory response, epithelial-mesenchymal transition (EMT) signatures, and other signatures, were activated in the PLXNC1 high-expression group (Figure 4A). Moreover, Kyoto Encyclopedia of Genes and Genomes (KEGG) pathway analysis revealed that gene sets up-regulated in the high PLXNC1 sub-group were enriched with represented signatures involved in tumor development and progression, such as the JAK-STAT signaling pathway, ECM-receptor interaction, and cAMP signaling pathway (Figure 4B). We then used the GSEA to explore the cancer hallmark pathway enrichment with extract statistical results. The clusterprofiler package (11) was used to construct the GSEA plot of the cumulative curve, and the results showed the top five significant enrichment pathways with statistically significant signatures (enrichment score > 0, *P* < 0.05, Figure 4C). Routinely, we selected the significantly dysregulated genes in the aforementioned signaling pathways for validation. The qRT-PCR results showed that overexpression of PLXNC1 significantly enhanced the EMT, IL-6/STAT3, and inflammatory response-related genes such as IGFBP3, IL6ST, KIF1B, and FPR1 (Figure 4D). These results demonstrated that PLXNC1 accelerated the cancer development and progression by activating the cancerous signaling pathways in GC cells.

### PLXNC1 Regulates IL6ST Expression at the DNA Level in GC Cells

**Figure 5:**
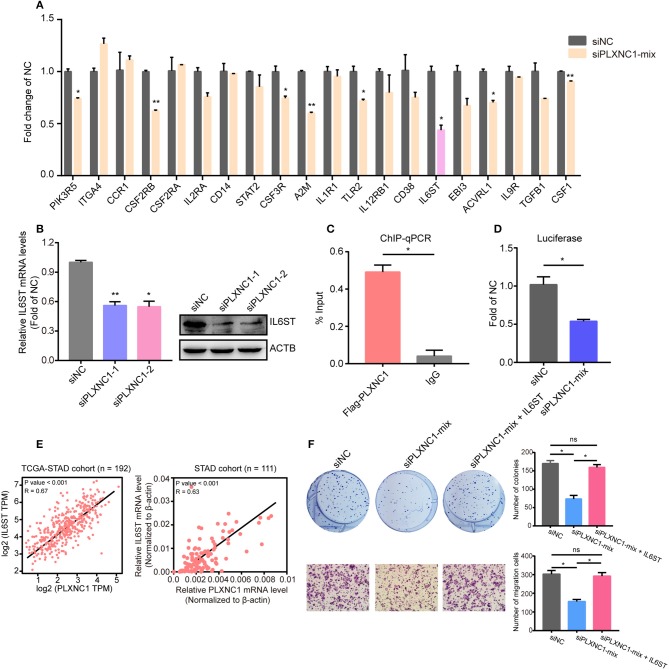
PLXNC1 controls IL6ST expression at the DNA level. **(A)** The relative mRNA levels of the IL-6/STAT3 pathway genes in AGS cells transfected with PLXNC1-mixed siRNAs and negative control siRNA. **(B)** IL6ST mRNA levers and protein levels in AGS cells transfected with PLXNC1 siRNAs and negative control. **(C)** ChIP-qPCR revealed the enrichment of PLXNC1 in IL6ST promoter in AGS cells. **(D)** The IL6ST promoter activity transfected with PLXNC1-mixed siRNAs and negative control siRNA in AGS cells. **(E)** Expressional correlation of PLXNC1 and IL6ST in GC tissues. **(F)** Colony and migration assays of AGS cells transfected with PLXNC1-mixed siRNAs, PLXNC1-mixed siRNAs plus IL6ST overexpression plasmids, or negative control. **(A–D,F)** Values represent the mean ± SEM, *n* = 3. *P < 0.05; **P < 0.01.

IL-6/STAT3 has been identified as a crucial pathway to accelerate GC progression (12, 13). Our previous studies highlighted that PLXNC1 activates IL-6/STAT3 signaling pathway in GC cells; however, the direct downstream targets of PLXNC1 still remain unclear. We first analyzed the expressional correlation of genes in this pathway with PLXNC1, and found the mRNA expression of 35 genes was significantly correlated with PLXNC1 (*R* ≥ 0.4), which elucidated the regulatory mechanism of PLXNC1 in the IL-6/STAT3 signaling pathway. Next, using the qRT-PCR method, we selected the top 20 genes in order to identify the potential regulation by PLXNC1, and found that knockdown of PLXNC1 could decrease the expression of genes such as CSF2RB (Figure 5A). Notably, knockdown of PLXNC1 could significantly diminish IL6ST mRNA levels (Figures 5A,B), while overexpression of PLXNC1 enhanced IL6ST mRNA levels (Figure 4D). These findings showed that IL6ST might be the direct downstream target of PLXNC1. IL6ST (also known as GP130) controls the IL-6/STAT3 signaling pathway and accelerates gastric tumorigenesis (14, 15). We performed ChIP-qPCR and found PLXNC1 was enriched on the IL6ST promoter (Figure 5C), further identifying the expressional control of IL6ST by PLXNC1 under a DNA lever. The dual-luciferase reporter assay also showed that knockdown of PLXNC1 decreased IL6ST promoter activity (Figure 5D); PLXNC1 expression was highly correlated with LI6ST expression in TCGA-STAD samples (left) and our internal GC samples (right; Figure 5E). Importantly, overexpression of IL6ST could rescue PLXNC1-deficient GC cell proliferation and migration (Figure 5F). Collectively, this data suggests IL6ST as a downstream target of PLXNC1 in GC.

## Discussion

An increasing number of studies have revealed the crucial regulatory roles of TFs in the manipulation of tumor-specific, addictive transcripts or cancer-related pathways, thus triggering carcinogenesis and promoting cancer development (16, 17). However, the complete function and clinical significance of TFs in GC remains unclear. In the present study, we systematically analyzed dysregulated TFs in GC and identified a critical role of transcriptional factor PLXNC1 in promoting GC progression, as well as the prognostic value of PLXNC1 in GC patients. We demonstrated that PLXNC1 was up-regulated in GC tissues, and GC patients with highly expressed PLXNC1 exhibited worse overall survival. Further studies identified that PLXNC1 promoted GC proliferation *in vitro* and *in vivo*, as well as migration *in vitro* by activating tumor-related pathways such as the IL-6/STAT3 signaling pathway.

Plexin C1 was first discovered in the nervous system and has been found to be associated with neuronal cell adhesion (18). Recent evidence shows that PLXNC1 participates in many crucial biological or disease processes. In papillary thyroid cancer (PTC), miR-4500 functions as a tumor suppressor by decreasing PLXNC1 expression, and knockdown of PLXNC1 represses colony formation, proliferation, invasiveness, and enhances apoptosis in PTC cells (19). In liver cancer, PLXNC1 marks epithelial phenotype of liver cancer cells and is significantly up-regulated in liver cancer tissues, which suggests the important roles of PLXNC1 in liver cancer (20). In the present study, we first reported the molecular function and clinical significance of PLXNC1, which served as an oncogene in promoting GC progression. PLXNC1 not only enhanced GC cell proliferation but also increased migration. High expression of PLXNC1 manipulated IL6ST expression at the DNA level and activated tumor-related pathways such as the IL-6/STAT3 pathway. This finding is in accordance with recent studies that have reported that PLXNC1 promotes acute inflammation (21). However, the whole genomic binding sites of PLXNC1 in GC remain unclear and need to be elucidated in further studies. Additionally, which factors control PLXNC1 expression in GC should be studied in more depth.

## Conclusion

Our study is the first to demonstrate that PLXNC1 is up-regulated and associated with poor survival in GC patients. PLXNC1 enhances the tumorigenesis and aggressiveness of GC cells through transcriptional activation of IL6ST and enhancement of the IL-6/STAT3 signaling pathway. These results reveal the crucial importance of PLXNC1 in GC progression, and suggest that the PLXNC1-IL6ST axis could be of potential value as a novel target of treatment for GC patients.

## Data Availability Statement

All datasets generated for this study are included in the article/Supplementary Material.

## Ethics Statement

The studies involving human participants were reviewed and approved by the Ethics Committee of Shanghai Medical College of Fudan University. The patients/participants provided their written informed consent to participate in this study. The animal study was reviewed and approved by the mouse experiments were conducted using the Guide for the Care and Use of Laboratory Animals of Fudan University and approved by the Committee on the Ethics and Welfare of Laboratory Animal Science of Fudan University.

## Author Contributions

CD and JW designed the study. JieC and HL acquired the data. JieC, HL, CD, JinC, and BS performed the analysis of data. JieC, HL, and CD wrote the paper with comments from all authors.

### Conflict of Interest

The authors declare that the research was conducted in the absence of any commercial or financial relationships that could be construed as a potential conflict of interest.
