# Supplementary material for: PLXNC1 Enhances Carcinogenesis Through Transcriptional Activation of IL6ST in Gastric Cancer
Source: Front Oncol. 2020 Feb 4;10:33. doi: 10.3389/fonc.2020.00033 (PMC7010712; doi:10.3389/fonc.2020.00033)
Supplement: Supplementary file 1 [file Table_1.docx]

**Supporting Materials**

**PLXNC1 Enhances Carcinogenesis through Transcriptional Activation of IL6ST in Gastric Cancer**

Jie Chen^1*^, Haining Liu^2*^, Jinggui Chen^1^, Bo Sun^1^, Jianghong Wu^1^, and Chunyan Du^1^

From the ^1^Department of Gastric Surgery, Fudan University Shanghai Cancer Center, Fudan University, 270 Dong An Road, Shanghai 200032, China;

^2^Department of Gastroenterology and Hepatology, Zhongshan Hospital, Fudan University, 180 Fenglin Road, Shanghai 200032, China.

^*^These authors have contributed equally to this work.

**Correspondence:**

Chunyan Du, M.D., E-mail: chunyanfudan@126.com; Department of Gastric Surgery, Fudan University Shanghai Cancer Center, Fudan University, 1205 Rm., 3# Bldg., 270 Dong An Rd., Shanghai 200032, China.

Or

Jianghong Wu, M.D., E-mail: elite53@163.com; Department of Gastric Surgery, Fudan University Shanghai Cancer Center, Fudan University, 1205 Rm., 3# Bldg., 270 Dong An Rd., Shanghai 200032, China.

**Supporting Figures**


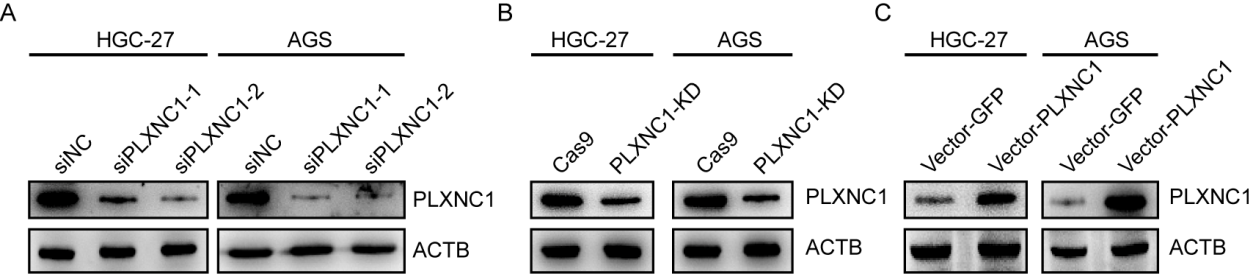


**Supporting Figure. S1.** Immunoblotting for PLXNC1 proteins in HGC-27 and AGS cells. (A) Immunoblotting for PLXNC1 proteins in HGC-27 and AGS cells after transfection with PLXNC1 siRNAs or the negative control (NC) siRNA. (B) Immunoblotting for PLXNC1 proteins in HGC-27 and AGS cells after infection with PLXNC1 knockdown sgRNA or control sgRNA lentivirus. (C) Immunoblotting for PLXNC1 proteins in HGC-27 and AGS cells after infection with PLXNC1 or GFP overexpression lentivirus. β-actin served as the loading control.

| **Supporting TABLE S1. Primers, sgRNA Sequences, and siRNA Sequences** | | | | |
| --- | --- | --- | --- | --- |
| Genes | | Sequences | | |
| PLXNC1-qF | | TTGGTGCCATTCGCTACAAAG | | |
| PLXNC1-qR | | TTTTGCTCCAGACGAAATATCCA | | |
| IGFBP3-qF | | AGAGCACAGATACCCAGAACT | | |
| IGFBP3-qR | | GGTGATTCAGTGTGTCTTCCATT | | |
| COL11A1-qF | | GTCCTCCAGGTCTACAAGGC | | |
| COL11A1-qR | | ACGGAACGGTAACATCAACATAG | | |
| FBLN5-qF | | CTACTCGAACCCCTACTCGAC | | |
| FBLN5-qR | | TCGTGGGATAGTTTGGAGCTG | | |
| LAMA2-qF | | GAACTGCTAGAATTTACCTCCGC | | |
| LAMA2-qR | | GCAAACATCATCAAGTCAGCATT | | |
| CSF2RB-qF | | AGCGGCTTCAGGACTCTTG | | |
| CSF2RB -qR | | CTGGGCATGAGGTGCTCTG | | |
| IL6ST-qF | | CGGACAGCTTGAACAGAATGT | | |
| IL6ST-qR | | ACCATCCCACTCACACCTCA | | |
| CXCL13-qF | | GCTTGAGGTGTAGATGTGTCC | | |
| CXCL13-qR | | CCCACGGGGCAAGATTTGAA | | |
| JUN-qF | | GAGCTGGAGCGCCTGATAAT | | |
| JUN-qR | | CCCTCCTGCTCATCTGTCAC | | |
| FPR1-qF | | GCTCCTCACATTGCCAGTTAT | | |
| FPR1-qR | | CGTTGGTCCAGGGCGAAAA | | |
| CCL7-qF | | CTTGCTCAGCCAGTTGGGAT | | |
| CCL7-qR | | GACAGTGGCTACTGGTGGTC | | |
| GPR183-qF | | ACTGGAGAATCGGAGATGCCT | | |
| GPR183-qR | | AATGAAGCGGTCAATACTCAGG | | |
| KIF1B-qF | | AAACAAGGGTAATTTGCGTGTGC | | |
| KIF1B-qR | | GTAACTGCCAACTTGGACAGAT | | |
| sgPLXNC1-1 | | GGAGATACCTTTGTAGCGAA | | |
| sgPLXNC1-2 | | GCCATAAACGGATGTCAGGT | | |
| siPLXNC1-1 | | CCATTCCGACCTGACATCCGTTTAT | | |
| siPLXNC1-2 | | CAGGCTTTACAGGTCTTCTACATTA | | |
| ChIP-qPCR-F | | CGCGTTACGGGAATCGCTA | | |
| ChIP-qPCR-R | | GGGACCTAGGCGCTGATTG | | |
| **Supporting TABLE S2. Antibodies Used in This Study** | | | | |
| Name | Company | | Catalog Number | Use |
| PLXNC1 | Abcam | | ab116070 | WB |
| ACTB | Sigma-Aldrich | | A2228 | WB |
| IL6ST | Abcam | | ab202850 | WB |
| Flag-tag | Cell signaling technology | | 14793 | ChIP |

**Supporting TABLE S3. The Differential Expressed TFs in TCGA-STAD Database**

| Ensemble number | logFC | P.Value | adj.P.Val |
| --- | --- | --- | --- |
| ENSG00000101057 | 2.8930 | 0.0000 | 0.0000 |
| ENSG00000125398 | 2.5264 | 0.0000 | 0.0000 |
| ENSG00000175832 | 2.3844 | 0.0000 | 0.0000 |
| ENSG00000180818 | 2.3750 | 0.0000 | 0.0000 |
| ENSG00000111206 | 2.2567 | 0.0000 | 0.0000 |
| ENSG00000050344 | 2.2028 | 0.0000 | 0.0000 |
| ENSG00000183734 | 2.1816 | 0.0000 | 0.0004 |
| ENSG00000101076 | 2.0484 | 0.0001 | 0.0011 |
| ENSG00000101412 | 1.9534 | 0.0000 | 0.0000 |
| ENSG00000124766 | 1.9214 | 0.0000 | 0.0000 |
| ENSG00000137309 | 1.8391 | 0.0000 | 0.0003 |
| ENSG00000029993 | 1.7903 | 0.0000 | 0.0000 |
| ENSG00000170689 | 1.6892 | 0.0000 | 0.0000 |
| ENSG00000113722 | 1.6663 | 0.0216 | 0.1014 |
| ENSG00000119547 | 1.6150 | 0.0000 | 0.0003 |
| ENSG00000105976 | 1.5999 | 0.0000 | 0.0000 |
| ENSG00000129654 | 1.5746 | 0.0000 | 0.0000 |
| ENSG00000118513 | 1.5007 | 0.0000 | 0.0000 |
| ENSG00000165556 | 1.4700 | 0.0014 | 0.0127 |
| ENSG00000157613 | 1.4667 | 0.0047 | 0.0332 |
| ENSG00000197905 | 1.4258 | 0.0000 | 0.0000 |
| ENSG00000054598 | 1.4096 | 0.0000 | 0.0001 |
| ENSG00000130589 | 1.4093 | 0.0000 | 0.0000 |
| ENSG00000179772 | 1.4023 | 0.0000 | 0.0000 |
| ENSG00000159184 | 1.3943 | 0.0001 | 0.0015 |
| ENSG00000139515 | 1.3796 | 0.0005 | 0.0054 |
| ENSG00000163435 | 1.3657 | 0.0039 | 0.0287 |
| ENSG00000100558 | 1.3551 | 0.0032 | 0.0243 |
| ENSG00000164749 | 1.3254 | 0.0029 | 0.0227 |
| ENSG00000115415 | 1.3240 | 0.0000 | 0.0001 |
| ENSG00000049768 | 1.3080 | 0.0000 | 0.0000 |
| ENSG00000124920 | 1.3026 | 0.0021 | 0.0175 |
| ENSG00000102554 | 1.2969 | 0.0103 | 0.0586 |
| ENSG00000051180 | 1.2894 | 0.0000 | 0.0000 |
| ENSG00000168062 | 1.2874 | 0.0001 | 0.0022 |
| ENSG00000135100 | 1.2639 | 0.0000 | 0.0001 |
| ENSG00000159216 | 1.2543 | 0.0000 | 0.0000 |
| ENSG00000144485 | 1.2503 | 0.0000 | 0.0001 |
| ENSG00000156127 | 1.2458 | 0.0002 | 0.0026 |
| ENSG00000024526 | 1.2355 | 0.0000 | 0.0000 |
| ENSG00000183742 | 1.2211 | 0.0000 | 0.0006 |
| ENSG00000106462 | 1.2163 | 0.0000 | 0.0000 |
| ENSG00000165244 | 1.2059 | 0.0000 | 0.0000 |
| ENSG00000077150 | 1.1851 | 0.0000 | 0.0000 |
| ENSG00000071564 | 1.1748 | 0.0000 | 0.0000 |
| ENSG00000125798 | 1.1712 | 0.0178 | 0.0881 |
| ENSG00000035499 | 1.1696 | 0.0000 | 0.0000 |
| ENSG00000129911 | 1.1657 | 0.0000 | 0.0000 |
| ENSG00000020633 | 1.1632 | 0.0001 | 0.0021 |
| ENSG00000185507 | 1.1625 | 0.0000 | 0.0001 |
| ENSG00000114554 | 1.1501 | 0.0000 | 0.0000 |
| ENSG00000081059 | 1.1175 | 0.0000 | 0.0009 |
| ENSG00000118707 | 1.1075 | 0.0000 | 0.0000 |
| ENSG00000009950 | 1.0998 | 0.0013 | 0.0118 |
| ENSG00000164078 | 1.0911 | 0.0024 | 0.0196 |
| ENSG00000175592 | 1.0873 | 0.0056 | 0.0377 |
| ENSG00000140968 | 1.0845 | 0.0016 | 0.0141 |
| ENSG00000112242 | 1.0810 | 0.0000 | 0.0000 |
| ENSG00000137166 | 1.0704 | 0.0000 | 0.0001 |
| ENSG00000196950 | 1.0679 | 0.0000 | 0.0000 |
| ENSG00000006047 | 1.0488 | 0.0001 | 0.0021 |
| ENSG00000004399 | 1.0459 | 0.0000 | 0.0002 |
| ENSG00000134954 | 1.0450 | 0.0000 | 0.0006 |
| ENSG00000137310 | 1.0409 | 0.0000 | 0.0000 |
| ENSG00000179344 | 1.0282 | 0.0160 | 0.0815 |
| ENSG00000010030 | 1.0280 | 0.0007 | 0.0074 |
| ENSG00000178860 | 1.0210 | 0.0000 | 0.0007 |
| ENSG00000104856 | 1.0205 | 0.0000 | 0.0001 |
| ENSG00000170608 | 1.0181 | 0.0526 | 0.1898 |
| ENSG00000067955 | 1.0154 | 0.0000 | 0.0000 |
| ENSG00000129173 | 1.0120 | 0.0000 | 0.0001 |
| ENSG00000171940 | 1.0054 | 0.0000 | 0.0001 |
| ENSG00000185404 | 0.9987 | 0.0000 | 0.0000 |
| ENSG00000120149 | 0.9972 | 0.0007 | 0.0071 |
| ENSG00000130675 | 0.9958 | 0.0000 | 0.0000 |
| ENSG00000134815 | 0.9941 | 0.0000 | 0.0000 |
| ENSG00000109685 | 0.9882 | 0.0000 | 0.0000 |
| ENSG00000179588 | 0.9822 | 0.0003 | 0.0042 |
| ENSG00000167771 | 0.9813 | 0.0000 | 0.0001 |
| ENSG00000164379 | 0.9775 | 0.0638 | 0.2172 |
| ENSG00000014164 | 0.9649 | 0.0000 | 0.0000 |
| ENSG00000111424 | 0.9555 | 0.0110 | 0.0616 |
| ENSG00000115956 | 0.9526 | 0.0057 | 0.0382 |
| ENSG00000066336 | 0.9415 | 0.0037 | 0.0271 |
| ENSG00000083307 | 0.9392 | 0.0008 | 0.0088 |
| ENSG00000184445 | 0.9375 | 0.0000 | 0.0000 |
| ENSG00000135373 | 0.9291 | 0.0558 | 0.1977 |
| ENSG00000160710 | 0.9273 | 0.0000 | 0.0000 |
| ENSG00000214022 | 0.9176 | 0.0000 | 0.0000 |
| ENSG00000029153 | 0.9173 | 0.0009 | 0.0092 |
| ENSG00000124226 | 0.9161 | 0.0000 | 0.0000 |
| ENSG00000177426 | 0.9140 | 0.0000 | 0.0000 |
| ENSG00000106546 | 0.9092 | 0.0000 | 0.0000 |
| ENSG00000059378 | 0.9049 | 0.0001 | 0.0014 |
| ENSG00000164104 | 0.9045 | 0.0000 | 0.0000 |
| ENSG00000163909 | 0.8993 | 0.0010 | 0.0102 |
| ENSG00000137203 | 0.8916 | 0.0023 | 0.0189 |
| ENSG00000171316 | 0.8915 | 0.0000 | 0.0000 |
| ENSG00000152804 | 0.8886 | 0.0001 | 0.0013 |
| ENSG00000204604 | 0.8884 | 0.0000 | 0.0000 |
| ENSG00000130827 | 0.8876 | 0.0000 | 0.0001 |
| ENSG00000196126 | 0.8851 | 0.0474 | 0.1765 |
| ENSG00000184967 | 0.8794 | 0.0000 | 0.0000 |
| ENSG00000124201 | 0.8788 | 0.0000 | 0.0000 |
| ENSG00000126456 | 0.8774 | 0.0000 | 0.0000 |
| ENSG00000103460 | 0.8770 | 0.0140 | 0.0736 |
| ENSG00000141448 | 0.8761 | 0.0557 | 0.1976 |
| ENSG00000137834 | 0.8715 | 0.0000 | 0.0004 |
| ENSG00000177485 | 0.8712 | 0.0000 | 0.0000 |
| ENSG00000129514 | 0.8700 | 0.0403 | 0.1576 |
| ENSG00000183779 | 0.8698 | 0.0070 | 0.0442 |
| ENSG00000007968 | 0.8584 | 0.0002 | 0.0025 |
| ENSG00000100426 | 0.8467 | 0.0000 | 0.0000 |
| ENSG00000107485 | 0.8465 | 0.0000 | 0.0003 |
| ENSG00000163013 | 0.8449 | 0.0000 | 0.0000 |
| ENSG00000126767 | 0.8416 | 0.0000 | 0.0000 |
| ENSG00000125618 | 0.8387 | 0.0000 | 0.0001 |
| ENSG00000108671 | 0.8258 | 0.0000 | 0.0000 |
| ENSG00000157827 | 0.8255 | 0.0000 | 0.0002 |
| ENSG00000179115 | 0.8225 | 0.0000 | 0.0000 |
| ENSG00000117595 | 0.8188 | 0.0252 | 0.1132 |
| ENSG00000149136 | 0.8144 | 0.0000 | 0.0000 |
| ENSG00000198176 | 0.8121 | 0.0000 | 0.0000 |
| ENSG00000170684 | 0.8085 | 0.0024 | 0.0193 |
| ENSG00000090447 | 0.8083 | 0.0000 | 0.0000 |
| ENSG00000105821 | 0.8064 | 0.0000 | 0.0000 |
| ENSG00000103495 | 0.8042 | 0.0000 | 0.0000 |
| ENSG00000198554 | 0.8000 | 0.0000 | 0.0000 |
| ENSG00000165684 | 0.7921 | 0.0000 | 0.0000 |
| ENSG00000203485 | 0.7871 | 0.0003 | 0.0037 |
| ENSG00000126003 | 0.7849 | 0.0001 | 0.0013 |
| ENSG00000100207 | 0.7839 | 0.0000 | 0.0000 |
| ENSG00000172818 | 0.7806 | 0.0396 | 0.1558 |
| ENSG00000185947 | 0.7799 | 0.0000 | 0.0000 |
| ENSG00000072310 | 0.7798 | 0.0000 | 0.0006 |
| ENSG00000167491 | 0.7744 | 0.0000 | 0.0000 |
| ENSG00000168298 | 0.7739 | 0.0031 | 0.0238 |
| ENSG00000178691 | 0.7728 | 0.0000 | 0.0000 |
| ENSG00000124126 | 0.7722 | 0.0014 | 0.0130 |
| ENSG00000104907 | 0.7687 | 0.0000 | 0.0000 |
| ENSG00000124216 | 0.7649 | 0.0012 | 0.0115 |
| ENSG00000116017 | 0.7643 | 0.0000 | 0.0002 |
| ENSG00000005801 | 0.7605 | 0.0000 | 0.0000 |
| ENSG00000143418 | 0.7582 | 0.0001 | 0.0015 |
| ENSG00000138795 | 0.7580 | 0.0000 | 0.0008 |
| ENSG00000160336 | 0.7569 | 0.0000 | 0.0000 |
| ENSG00000139083 | 0.7528 | 0.0000 | 0.0000 |
| ENSG00000177732 | 0.7516 | 0.0004 | 0.0049 |
| ENSG00000125347 | 0.7512 | 0.0084 | 0.0506 |
| ENSG00000186416 | 0.7487 | 0.0000 | 0.0000 |
| ENSG00000136574 | 0.7479 | 0.1685 | 0.4010 |
| ENSG00000141510 | 0.7465 | 0.0003 | 0.0033 |
| ENSG00000204335 | 0.7455 | 0.0310 | 0.1307 |
| ENSG00000157657 | 0.7419 | 0.0000 | 0.0002 |
| ENSG00000197961 | 0.7410 | 0.0000 | 0.0000 |
| ENSG00000101126 | 0.7378 | 0.0000 | 0.0000 |
| ENSG00000188290 | 0.7306 | 0.0022 | 0.0186 |
| ENSG00000183340 | 0.7293 | 0.0000 | 0.0000 |
| ENSG00000105722 | 0.7243 | 0.0002 | 0.0030 |
| ENSG00000196793 | 0.7242 | 0.0000 | 0.0001 |
| ENSG00000161405 | 0.7208 | 0.0233 | 0.1068 |
| ENSG00000148229 | 0.7196 | 0.0000 | 0.0000 |
| ENSG00000196233 | 0.7170 | 0.0002 | 0.0023 |
| ENSG00000205903 | 0.7167 | 0.0000 | 0.0001 |
| ENSG00000127337 | 0.7167 | 0.0000 | 0.0001 |
| ENSG00000196498 | 0.7138 | 0.0001 | 0.0012 |
| ENSG00000104897 | 0.7095 | 0.0000 | 0.0000 |
| ENSG00000127152 | 0.7089 | 0.0002 | 0.0028 |
| ENSG00000102034 | 0.7069 | 0.0025 | 0.0199 |
| ENSG00000167635 | 0.7058 | 0.0000 | 0.0000 |
| ENSG00000170561 | 0.7032 | 0.0836 | 0.2593 |
| ENSG00000133740 | 0.6981 | 0.0000 | 0.0000 |
| ENSG00000162702 | 0.6971 | 0.0000 | 0.0000 |
| ENSG00000104881 | 0.6957 | 0.0667 | 0.2236 |
| ENSG00000107929 | 0.6915 | 0.0000 | 0.0000 |
| ENSG00000172216 | 0.6893 | 0.0032 | 0.0246 |
| ENSG00000167157 | 0.6863 | 0.0259 | 0.1154 |
| ENSG00000143190 | 0.6845 | 0.0000 | 0.0000 |
| ENSG00000122034 | 0.6836 | 0.0000 | 0.0006 |
| ENSG00000172262 | 0.6804 | 0.0000 | 0.0000 |
| ENSG00000096696 | 0.6803 | 0.1984 | 0.4412 |
| ENSG00000106948 | 0.6793 | 0.0060 | 0.0397 |
| ENSG00000108064 | 0.6792 | 0.0000 | 0.0000 |
| ENSG00000197279 | 0.6759 | 0.0001 | 0.0011 |
| ENSG00000104885 | 0.6733 | 0.0001 | 0.0018 |
| ENSG00000138385 | 0.6723 | 0.0000 | 0.0002 |
| ENSG00000170581 | 0.6704 | 0.0002 | 0.0028 |
| ENSG00000143067 | 0.6702 | 0.0000 | 0.0000 |
| ENSG00000028310 | 0.6698 | 0.0000 | 0.0000 |
| ENSG00000167962 | 0.6695 | 0.0000 | 0.0001 |
| ENSG00000196247 | 0.6680 | 0.0000 | 0.0000 |
| ENSG00000173473 | 0.6627 | 0.0000 | 0.0000 |
| ENSG00000155506 | 0.6600 | 0.0000 | 0.0001 |
| ENSG00000129534 | 0.6598 | 0.0000 | 0.0001 |
| ENSG00000020256 | 0.6594 | 0.0000 | 0.0000 |
| ENSG00000146757 | 0.6581 | 0.0009 | 0.0089 |
| ENSG00000113716 | 0.6556 | 0.0000 | 0.0000 |
| ENSG00000100105 | 0.6535 | 0.0001 | 0.0014 |
| ENSG00000143390 | 0.6525 | 0.0001 | 0.0010 |
| ENSG00000158545 | 0.6511 | 0.0000 | 0.0000 |
| ENSG00000080603 | 0.6483 | 0.0000 | 0.0002 |
| ENSG00000196576 | 0.6475 | 0.0008 | 0.0083 |
| ENSG00000126464 | 0.6457 | 0.0001 | 0.0019 |
| ENSG00000171467 | 0.6445 | 0.0000 | 0.0001 |
| ENSG00000173145 | 0.6425 | 0.0000 | 0.0003 |
| ENSG00000204366 | 0.6422 | 0.0002 | 0.0024 |
| ENSG00000142409 | 0.6408 | 0.0001 | 0.0016 |
| ENSG00000136603 | 0.6396 | 0.0002 | 0.0031 |
| ENSG00000169136 | 0.6354 | 0.0093 | 0.0547 |
| ENSG00000124795 | 0.6322 | 0.0000 | 0.0000 |
| ENSG00000137492 | 0.6298 | 0.0001 | 0.0010 |
| ENSG00000185122 | 0.6288 | 0.0000 | 0.0001 |
| ENSG00000001167 | 0.6265 | 0.0000 | 0.0000 |
| ENSG00000137504 | 0.6251 | 0.0001 | 0.0017 |
| ENSG00000198502 | 0.6234 | 0.1905 | 0.4307 |
| ENSG00000163132 | 0.6203 | 0.0016 | 0.0141 |
| ENSG00000198466 | 0.6198 | 0.0000 | 0.0002 |
| ENSG00000198538 | 0.6170 | 0.0000 | 0.0008 |
| ENSG00000170802 | 0.6169 | 0.0000 | 0.0002 |
| ENSG00000125850 | 0.6158 | 0.0431 | 0.1653 |
| ENSG00000161813 | 0.6118 | 0.0000 | 0.0001 |
| ENSG00000167548 | 0.6118 | 0.0009 | 0.0091 |
| ENSG00000143379 | 0.6117 | 0.0000 | 0.0000 |
| ENSG00000181135 | 0.6110 | 0.0000 | 0.0000 |
| ENSG00000149480 | 0.6095 | 0.0000 | 0.0002 |
| ENSG00000141994 | 0.6066 | 0.0000 | 0.0000 |
| ENSG00000179886 | 0.6065 | 0.0000 | 0.0000 |
| ENSG00000152926 | 0.6058 | 0.0097 | 0.0564 |
| ENSG00000185730 | 0.6046 | 0.0000 | 0.0000 |
| ENSG00000123095 | 0.6046 | 0.0172 | 0.0857 |
| ENSG00000122386 | 0.6036 | 0.0000 | 0.0001 |
| ENSG00000108511 | 0.6029 | 0.0439 | 0.1672 |
| ENSG00000189120 | 0.6029 | 0.0219 | 0.1024 |
| ENSG00000213676 | 0.6025 | 0.0000 | 0.0003 |
| ENSG00000185238 | 0.6023 | 0.0000 | 0.0000 |
| ENSG00000198026 | 0.6006 | 0.0000 | 0.0000 |
| ENSG00000170448 | 0.5992 | 0.0000 | 0.0000 |
| ENSG00000197170 | 0.5990 | 0.0000 | 0.0000 |
| ENSG00000167395 | 0.5943 | 0.0000 | 0.0002 |
| ENSG00000128604 | 0.5919 | 0.0056 | 0.0375 |
| ENSG00000143157 | 0.5908 | 0.0000 | 0.0001 |
| ENSG00000138073 | 0.5878 | 0.0007 | 0.0073 |
| ENSG00000101216 | 0.5844 | 0.0000 | 0.0000 |
| ENSG00000177425 | 0.5819 | 0.0002 | 0.0032 |
| ENSG00000205250 | 0.5790 | 0.0000 | 0.0001 |
| ENSG00000115568 | 0.5790 | 0.0000 | 0.0004 |
| ENSG00000153048 | 0.5763 | 0.0016 | 0.0141 |
| ENSG00000142528 | 0.5760 | 0.0000 | 0.0000 |
| ENSG00000162813 | 0.5671 | 0.0063 | 0.0410 |
| ENSG00000112365 | 0.5668 | 0.0000 | 0.0000 |
| ENSG00000180884 | 0.5666 | 0.0000 | 0.0001 |
| ENSG00000158773 | 0.5663 | 0.0000 | 0.0009 |
| ENSG00000136870 | 0.5654 | 0.0000 | 0.0002 |
| ENSG00000100644 | 0.5633 | 0.0018 | 0.0156 |
| ENSG00000120798 | 0.5618 | 0.0000 | 0.0002 |
| ENSG00000142684 | 0.5604 | 0.0001 | 0.0015 |
| ENSG00000182944 | 0.5597 | 0.0000 | 0.0000 |
| ENSG00000143373 | 0.5580 | 0.0000 | 0.0001 |
| ENSG00000144852 | 0.5578 | 0.1266 | 0.3401 |
| ENSG00000141298 | 0.5567 | 0.0000 | 0.0000 |
| ENSG00000102870 | 0.5563 | 0.0005 | 0.0053 |
| ENSG00000143842 | 0.5558 | 0.0003 | 0.0043 |
| ENSG00000181638 | 0.5541 | 0.0000 | 0.0000 |
| ENSG00000198546 | 0.5541 | 0.0003 | 0.0034 |
| ENSG00000160917 | 0.5511 | 0.0000 | 0.0002 |
| ENSG00000183431 | 0.5497 | 0.0000 | 0.0000 |
| ENSG00000175213 | 0.5482 | 0.0000 | 0.0001 |
| ENSG00000124151 | 0.5482 | 0.0011 | 0.0107 |
| ENSG00000173404 | 0.5479 | 0.2747 | 0.5371 |
| ENSG00000116120 | 0.5469 | 0.0000 | 0.0002 |
| ENSG00000130749 | 0.5468 | 0.0000 | 0.0000 |
| ENSG00000167625 | 0.5467 | 0.0000 | 0.0001 |
| ENSG00000168883 | 0.5463 | 0.0000 | 0.0000 |
| ENSG00000153879 | 0.5446 | 0.0007 | 0.0074 |
| ENSG00000189403 | 0.5440 | 0.0000 | 0.0001 |
| ENSG00000198429 | 0.5411 | 0.0004 | 0.0049 |
| ENSG00000163516 | 0.5409 | 0.0000 | 0.0001 |
| ENSG00000106261 | 0.5407 | 0.0022 | 0.0182 |
| ENSG00000104472 | 0.5390 | 0.0000 | 0.0001 |
| ENSG00000158717 | 0.5381 | 0.0010 | 0.0103 |
| ENSG00000083635 | 0.5366 | 0.0000 | 0.0000 |
| ENSG00000214717 | 0.5365 | 0.0006 | 0.0064 |
| ENSG00000158711 | 0.5358 | 0.0000 | 0.0009 |
| ENSG00000164916 | 0.5354 | 0.0028 | 0.0220 |
| ENSG00000171163 | 0.5340 | 0.0002 | 0.0029 |
| ENSG00000163159 | 0.5283 | 0.0000 | 0.0002 |
| ENSG00000169926 | 0.5270 | 0.0013 | 0.0124 |
| ENSG00000163872 | 0.5266 | 0.0003 | 0.0035 |
| ENSG00000125817 | 0.5255 | 0.0022 | 0.0182 |
| ENSG00000126368 | 0.5250 | 0.0174 | 0.0867 |
| ENSG00000153147 | 0.5247 | 0.0004 | 0.0043 |
| ENSG00000183309 | 0.5212 | 0.0000 | 0.0004 |
| ENSG00000182872 | 0.5210 | 0.0000 | 0.0002 |
| ENSG00000079263 | 0.5204 | 0.0210 | 0.0994 |
| ENSG00000124664 | 0.5194 | 0.3204 | 0.5625 |
| ENSG00000126603 | 0.5186 | 0.0315 | 0.1321 |
| ENSG00000170485 | 0.5170 | 0.0243 | 0.1103 |
| ENSG00000141568 | 0.5170 | 0.0000 | 0.0001 |
| ENSG00000196812 | 0.5155 | 0.0000 | 0.0009 |
| ENSG00000176248 | 0.5143 | 0.0000 | 0.0000 |
| ENSG00000198522 | 0.5142 | 0.0000 | 0.0000 |
| ENSG00000164050 | 0.5111 | 0.0184 | 0.0899 |
| ENSG00000076108 | 0.5107 | 0.0008 | 0.0087 |
| ENSG00000139842 | 0.5100 | 0.0000 | 0.0001 |
| ENSG00000139746 | 0.5069 | 0.0002 | 0.0029 |
| ENSG00000109320 | 0.5057 | 0.0000 | 0.0005 |
| ENSG00000177853 | 0.5032 | 0.0007 | 0.0076 |
| ENSG00000132005 | 0.5029 | 0.0000 | 0.0001 |
| ENSG00000164683 | 0.5026 | 0.0002 | 0.0025 |
| ENSG00000166716 | 0.5011 | 0.0001 | 0.0018 |
| ENSG00000204569 | 0.5009 | 0.0000 | 0.0004 |
| ENSG00000135457 | 0.4989 | 0.0000 | 0.0001 |
| ENSG00000211899 | 0.4978 | 0.6152 | 0.8229 |
| ENSG00000169957 | 0.4975 | 0.0048 | 0.0333 |
| ENSG00000132773 | 0.4936 | 0.0000 | 0.0008 |
| ENSG00000081721 | 0.4926 | 0.0000 | 0.0006 |
| ENSG00000173545 | 0.4918 | 0.0006 | 0.0066 |
| ENSG00000149016 | 0.4902 | 0.0000 | 0.0000 |
| ENSG00000153207 | 0.4899 | 0.0003 | 0.0041 |
| ENSG00000105556 | 0.4893 | 0.0003 | 0.0034 |
| ENSG00000180346 | 0.4888 | 0.0005 | 0.0057 |
| ENSG00000213347 | 0.4883 | 0.0001 | 0.0010 |
| ENSG00000126561 | 0.4883 | 0.0039 | 0.0285 |
| ENSG00000100281 | 0.4877 | 0.0000 | 0.0000 |
| ENSG00000218891 | 0.4871 | 0.0099 | 0.0570 |
| ENSG00000198824 | 0.4870 | 0.0000 | 0.0006 |
| ENSG00000137074 | 0.4867 | 0.0000 | 0.0000 |
| ENSG00000101096 | 0.4858 | 0.0068 | 0.0435 |
| ENSG00000180257 | 0.4825 | 0.0002 | 0.0023 |
| ENSG00000198556 | 0.4805 | 0.0000 | 0.0004 |
| ENSG00000156983 | 0.4798 | 0.0000 | 0.0002 |
| ENSG00000189190 | 0.4791 | 0.0002 | 0.0024 |
| ENSG00000197008 | 0.4787 | 0.0000 | 0.0006 |
| ENSG00000132170 | 0.4770 | 0.1114 | 0.3129 |
| ENSG00000100403 | 0.4763 | 0.0004 | 0.0047 |
| ENSG00000197114 | 0.4744 | 0.0000 | 0.0001 |
| ENSG00000065548 | 0.4738 | 0.0004 | 0.0049 |
| ENSG00000010539 | 0.4737 | 0.0000 | 0.0000 |
| ENSG00000147180 | 0.4725 | 0.0005 | 0.0056 |
| ENSG00000185252 | 0.4721 | 0.0002 | 0.0024 |
| ENSG00000121068 | 0.4717 | 0.0036 | 0.0269 |
| ENSG00000134323 | 0.4717 | 0.0246 | 0.1112 |
| ENSG00000125352 | 0.4712 | 0.0002 | 0.0030 |
| ENSG00000140265 | 0.4710 | 0.0000 | 0.0008 |
| ENSG00000130584 | 0.4688 | 0.0001 | 0.0022 |
| ENSG00000169016 | 0.4667 | 0.0000 | 0.0001 |
| ENSG00000114315 | 0.4612 | 0.0349 | 0.1422 |
| ENSG00000136243 | 0.4612 | 0.0001 | 0.0013 |
| ENSG00000132967 | 0.4600 | 0.0066 | 0.0422 |
| ENSG00000010244 | 0.4593 | 0.0000 | 0.0003 |
| ENSG00000170322 | 0.4585 | 0.0003 | 0.0037 |
| ENSG00000135111 | 0.4565 | 0.1286 | 0.3436 |
| ENSG00000166526 | 0.4565 | 0.0002 | 0.0032 |
| ENSG00000197608 | 0.4563 | 0.0010 | 0.0096 |
| ENSG00000162419 | 0.4558 | 0.0000 | 0.0000 |
| ENSG00000187837 | 0.4555 | 0.1438 | 0.3692 |
| ENSG00000136040 | 0.4553 | 0.0052 | 0.0353 |
| ENSG00000182986 | 0.4532 | 0.0004 | 0.0047 |
| ENSG00000136237 | 0.4530 | 0.0017 | 0.0153 |
| ENSG00000131061 | 0.4527 | 0.0000 | 0.0000 |
| ENSG00000102974 | 0.4519 | 0.0000 | 0.0000 |
| ENSG00000180035 | 0.4516 | 0.0030 | 0.0233 |
| ENSG00000056277 | 0.4514 | 0.0001 | 0.0019 |
| ENSG00000196453 | 0.4491 | 0.0004 | 0.0047 |
| ENSG00000147789 | 0.4483 | 0.0000 | 0.0000 |
| ENSG00000025293 | 0.4479 | 0.0001 | 0.0021 |
| ENSG00000153975 | 0.4453 | 0.0000 | 0.0003 |
| ENSG00000117748 | 0.4448 | 0.0009 | 0.0089 |
| ENSG00000105732 | 0.4447 | 0.0002 | 0.0030 |
| ENSG00000183495 | 0.4441 | 0.0021 | 0.0176 |
| ENSG00000198169 | 0.4394 | 0.0033 | 0.0249 |
| ENSG00000188342 | 0.4391 | 0.0000 | 0.0006 |
| ENSG00000181220 | 0.4361 | 0.0000 | 0.0001 |
| ENSG00000151657 | 0.4359 | 0.0000 | 0.0000 |
| ENSG00000058673 | 0.4341 | 0.0028 | 0.0219 |
| ENSG00000116132 | 0.4341 | 0.0327 | 0.1357 |
| ENSG00000188295 | 0.4339 | 0.0004 | 0.0043 |
| ENSG00000165066 | 0.4332 | 0.3143 | 0.5625 |
| ENSG00000148187 | 0.4324 | 0.0000 | 0.0005 |
| ENSG00000166860 | 0.4318 | 0.0003 | 0.0041 |
| ENSG00000164920 | 0.4315 | 0.0463 | 0.1736 |
| ENSG00000173153 | 0.4312 | 0.0478 | 0.1776 |
| ENSG00000167394 | 0.4292 | 0.0000 | 0.0002 |
| ENSG00000166949 | 0.4281 | 0.0079 | 0.0483 |
| ENSG00000179195 | 0.4279 | 0.0030 | 0.0236 |
| ENSG00000204946 | 0.4255 | 0.0000 | 0.0009 |
| ENSG00000157933 | 0.4253 | 0.0343 | 0.1405 |
| ENSG00000179943 | 0.4252 | 0.0000 | 0.0005 |
| ENSG00000120963 | 0.4232 | 0.0010 | 0.0096 |
| ENSG00000107404 | 0.4221 | 0.0150 | 0.0777 |
| ENSG00000170265 | 0.4213 | 0.0003 | 0.0041 |
| ENSG00000168610 | 0.4202 | 0.0013 | 0.0123 |
| ENSG00000165512 | 0.4195 | 0.0070 | 0.0441 |
| ENSG00000171295 | 0.4192 | 0.0088 | 0.0523 |
| ENSG00000181896 | 0.4151 | 0.0011 | 0.0106 |
| ENSG00000140548 | 0.4140 | 0.0152 | 0.0782 |
| ENSG00000177888 | 0.4116 | 0.0004 | 0.0043 |
| ENSG00000124813 | 0.4102 | 0.0139 | 0.0733 |
| ENSG00000105127 | 0.4096 | 0.0000 | 0.0002 |
| ENSG00000147118 | 0.4087 | 0.0004 | 0.0045 |
| ENSG00000205213 | 0.4080 | 0.0779 | 0.2480 |
| ENSG00000157259 | 0.4078 | 0.0115 | 0.0637 |
| ENSG00000167967 | 0.4055 | 0.0000 | 0.0002 |
| ENSG00000096654 | 0.4050 | 0.0006 | 0.0066 |
| ENSG00000140382 | 0.4044 | 0.0002 | 0.0026 |
| ENSG00000171161 | 0.4033 | 0.0006 | 0.0062 |
| ENSG00000167840 | 0.4032 | 0.0006 | 0.0069 |
| ENSG00000158805 | 0.4029 | 0.0035 | 0.0260 |
| ENSG00000198517 | 0.4015 | 0.0214 | 0.1006 |
| ENSG00000148737 | 0.4011 | 0.0143 | 0.0750 |
| ENSG00000135899 | 0.3996 | 0.0007 | 0.0075 |
| ENSG00000088876 | 0.3991 | 0.0006 | 0.0064 |
| ENSG00000134758 | 0.3987 | 0.0001 | 0.0016 |
| ENSG00000197483 | 0.3986 | 0.0001 | 0.0021 |
| ENSG00000116580 | 0.3985 | 0.0004 | 0.0044 |
| ENSG00000161940 | 0.3978 | 0.0089 | 0.0527 |
| ENSG00000131759 | 0.3965 | 0.0626 | 0.2143 |
| ENSG00000124496 | 0.3950 | 0.0114 | 0.0633 |
| ENSG00000125482 | 0.3949 | 0.0004 | 0.0048 |
| ENSG00000152518 | 0.3920 | 0.0289 | 0.1244 |
| ENSG00000204576 | 0.3913 | 0.0003 | 0.0036 |
| ENSG00000213928 | 0.3911 | 0.0077 | 0.0476 |
| ENSG00000182903 | 0.3898 | 0.0050 | 0.0344 |
| ENSG00000182979 | 0.3897 | 0.0011 | 0.0108 |
| ENSG00000166261 | 0.3889 | 0.0001 | 0.0020 |
| ENSG00000185591 | 0.3875 | 0.0044 | 0.0315 |
| ENSG00000181472 | 0.3872 | 0.0024 | 0.0194 |
| ENSG00000213020 | 0.3830 | 0.0001 | 0.0013 |
| ENSG00000124782 | 0.3823 | 0.0256 | 0.1143 |
| ENSG00000185811 | 0.3811 | 0.1597 | 0.3900 |
| ENSG00000115816 | 0.3789 | 0.0005 | 0.0062 |
| ENSG00000179456 | 0.3784 | 0.0146 | 0.0762 |
| ENSG00000153922 | 0.3778 | 0.0019 | 0.0161 |
| ENSG00000071655 | 0.3777 | 0.0011 | 0.0110 |
| ENSG00000043514 | 0.3777 | 0.0005 | 0.0061 |
| ENSG00000096401 | 0.3769 | 0.0001 | 0.0018 |
| ENSG00000119574 | 0.3763 | 0.0004 | 0.0052 |
| ENSG00000197265 | 0.3763 | 0.0089 | 0.0526 |
| ENSG00000006194 | 0.3753 | 0.0002 | 0.0029 |
| ENSG00000108312 | 0.3708 | 0.0007 | 0.0074 |
| ENSG00000120690 | 0.3692 | 0.0045 | 0.0318 |
| ENSG00000064933 | 0.3687 | 0.0012 | 0.0114 |
| ENSG00000124256 | 0.3687 | 0.0900 | 0.2721 |
| ENSG00000130803 | 0.3681 | 0.0000 | 0.0008 |
| ENSG00000065978 | 0.3676 | 0.0023 | 0.0189 |
| ENSG00000146587 | 0.3676 | 0.0079 | 0.0485 |
| ENSG00000122482 | 0.3669 | 0.0009 | 0.0091 |
| ENSG00000186230 | 0.3651 | 0.0003 | 0.0037 |
| ENSG00000198464 | 0.3641 | 0.0018 | 0.0158 |
| ENSG00000197362 | 0.3637 | 0.0010 | 0.0096 |
| ENSG00000056586 | 0.3634 | 0.0094 | 0.0551 |
| ENSG00000070476 | 0.3629 | 0.0022 | 0.0186 |
| ENSG00000025434 | 0.3627 | 0.1488 | 0.3772 |
| ENSG00000127481 | 0.3575 | 0.0197 | 0.0948 |
| ENSG00000178177 | 0.3573 | 0.0075 | 0.0466 |
| ENSG00000095951 | 0.3566 | 0.0091 | 0.0537 |
| ENSG00000123268 | 0.3565 | 0.0010 | 0.0099 |
| ENSG00000156853 | 0.3539 | 0.0065 | 0.0418 |
| ENSG00000167685 | 0.3537 | 0.0075 | 0.0465 |
| ENSG00000127124 | 0.3532 | 0.0021 | 0.0179 |
| ENSG00000204519 | 0.3526 | 0.0003 | 0.0041 |
| ENSG00000085276 | 0.3519 | 0.0885 | 0.2692 |
| ENSG00000163565 | 0.3510 | 0.1129 | 0.3158 |
| ENSG00000067066 | 0.3502 | 0.0067 | 0.0430 |
| ENSG00000118412 | 0.3499 | 0.0104 | 0.0591 |
| ENSG00000186130 | 0.3488 | 0.0002 | 0.0025 |
| ENSG00000108094 | 0.3486 | 0.0004 | 0.0052 |
| ENSG00000162664 | 0.3469 | 0.0012 | 0.0114 |
| ENSG00000184110 | 0.3442 | 0.0074 | 0.0460 |
| ENSG00000172845 | 0.3428 | 0.0042 | 0.0303 |
| ENSG00000116833 | 0.3423 | 0.1082 | 0.3070 |
| ENSG00000181827 | 0.3412 | 0.0125 | 0.0680 |
| ENSG00000162676 | 0.3400 | 0.0621 | 0.2129 |
| ENSG00000101190 | 0.3391 | 0.0382 | 0.1517 |
| ENSG00000126746 | 0.3389 | 0.0015 | 0.0136 |
| ENSG00000173041 | 0.3386 | 0.0050 | 0.0343 |
| ENSG00000119401 | 0.3377 | 0.0015 | 0.0134 |
| ENSG00000169981 | 0.3370 | 0.0001 | 0.0012 |
| ENSG00000112685 | 0.3368 | 0.0005 | 0.0060 |
| ENSG00000197162 | 0.3357 | 0.0010 | 0.0098 |
| ENSG00000105866 | 0.3356 | 0.0125 | 0.0680 |
| ENSG00000166188 | 0.3343 | 0.0155 | 0.0796 |
| ENSG00000171466 | 0.3324 | 0.0054 | 0.0366 |
| ENSG00000144161 | 0.3320 | 0.0008 | 0.0081 |
| ENSG00000184897 | 0.3318 | 0.0459 | 0.1725 |
| ENSG00000197037 | 0.3315 | 0.0024 | 0.0196 |
| ENSG00000139613 | 0.3313 | 0.0132 | 0.0705 |
| ENSG00000163170 | 0.3312 | 0.0451 | 0.1705 |
| ENSG00000129071 | 0.3284 | 0.0071 | 0.0445 |
| ENSG00000175197 | 0.3276 | 0.0388 | 0.1534 |
| ENSG00000161202 | 0.3271 | 0.0201 | 0.0961 |
| ENSG00000134107 | 0.3263 | 0.2013 | 0.4450 |
| ENSG00000105559 | 0.3262 | 0.1752 | 0.4095 |
| ENSG00000086589 | 0.3260 | 0.0002 | 0.0029 |
| ENSG00000111145 | 0.3257 | 0.0763 | 0.2449 |
| ENSG00000169951 | 0.3247 | 0.0048 | 0.0334 |
| ENSG00000196652 | 0.3243 | 0.0019 | 0.0162 |
| ENSG00000139624 | 0.3233 | 0.0004 | 0.0044 |
| ENSG00000085644 | 0.3232 | 0.0012 | 0.0116 |
| ENSG00000036549 | 0.3219 | 0.0059 | 0.0389 |
| ENSG00000186376 | 0.3207 | 0.0050 | 0.0343 |
| ENSG00000166925 | 0.3203 | 0.0400 | 0.1570 |
| ENSG00000160352 | 0.3197 | 0.0601 | 0.2081 |
| ENSG00000116809 | 0.3190 | 0.0007 | 0.0075 |
| ENSG00000174197 | 0.3157 | 0.0055 | 0.0368 |
| ENSG00000186566 | 0.3157 | 0.0056 | 0.0377 |
| ENSG00000056097 | 0.3145 | 0.0074 | 0.0458 |
| ENSG00000164631 | 0.3138 | 0.0120 | 0.0658 |
| ENSG00000011451 | 0.3133 | 0.0293 | 0.1257 |
| ENSG00000176182 | 0.3131 | 0.0021 | 0.0180 |
| ENSG00000175550 | 0.3127 | 0.0309 | 0.1305 |
| ENSG00000196670 | 0.3108 | 0.0203 | 0.0969 |
| ENSG00000196323 | 0.3093 | 0.0073 | 0.0457 |
| ENSG00000118217 | 0.3089 | 0.0007 | 0.0076 |
| ENSG00000168724 | 0.3065 | 0.0061 | 0.0398 |
| ENSG00000029363 | 0.3053 | 0.0063 | 0.0410 |
| ENSG00000112200 | 0.3051 | 0.0061 | 0.0398 |
| ENSG00000083168 | 0.3032 | 0.0084 | 0.0509 |
| ENSG00000163795 | 0.3032 | 0.0164 | 0.0830 |
| ENSG00000158290 | 0.3028 | 0.0956 | 0.2831 |
| ENSG00000148337 | 0.3026 | 0.0224 | 0.1041 |
| ENSG00000198455 | 0.3008 | 0.0077 | 0.0474 |
| ENSG00000189180 | 0.3005 | 0.0151 | 0.0781 |
| ENSG00000197714 | 0.2999 | 0.0969 | 0.2857 |
| ENSG00000076770 | 0.2996 | 0.0925 | 0.2770 |
| ENSG00000173039 | 0.2978 | 0.0037 | 0.0274 |
| ENSG00000125812 | 0.2970 | 0.0168 | 0.0844 |
| ENSG00000197782 | 0.2959 | 0.0041 | 0.0294 |
| ENSG00000064490 | 0.2915 | 0.0181 | 0.0891 |
| ENSG00000136770 | 0.2881 | 0.0421 | 0.1625 |
| ENSG00000142396 | 0.2875 | 0.0011 | 0.0105 |
| ENSG00000079432 | 0.2875 | 0.0710 | 0.2333 |
| ENSG00000124177 | 0.2874 | 0.0577 | 0.2020 |
| ENSG00000196693 | 0.2871 | 0.0182 | 0.0894 |
| ENSG00000132604 | 0.2867 | 0.0036 | 0.0268 |
| ENSG00000108788 | 0.2858 | 0.1014 | 0.2947 |
| ENSG00000104064 | 0.2854 | 0.0001 | 0.0016 |
| ENSG00000100811 | 0.2854 | 0.0000 | 0.0004 |
| ENSG00000066136 | 0.2852 | 0.0000 | 0.0008 |
| ENSG00000196757 | 0.2829 | 0.0422 | 0.1627 |
| ENSG00000126804 | 0.2815 | 0.0243 | 0.1102 |
| ENSG00000143578 | 0.2811 | 0.1646 | 0.3958 |
| ENSG00000173575 | 0.2809 | 0.0526 | 0.1897 |
| ENSG00000162924 | 0.2806 | 0.0166 | 0.0836 |
| ENSG00000127081 | 0.2801 | 0.0041 | 0.0294 |
| ENSG00000168214 | 0.2799 | 0.0380 | 0.1512 |
| ENSG00000181315 | 0.2791 | 0.0051 | 0.0349 |
| ENSG00000204611 | 0.2789 | 0.0003 | 0.0041 |
| ENSG00000180357 | 0.2789 | 0.0256 | 0.1144 |
| ENSG00000063587 | 0.2786 | 0.0500 | 0.1833 |
| ENSG00000011243 | 0.2775 | 0.0117 | 0.0645 |
| ENSG00000147124 | 0.2773 | 0.0017 | 0.0150 |
| ENSG00000086504 | 0.2772 | 0.0319 | 0.1331 |
| ENSG00000143442 | 0.2772 | 0.0559 | 0.1980 |
| ENSG00000124459 | 0.2771 | 0.0080 | 0.0490 |
| ENSG00000140262 | 0.2756 | 0.0452 | 0.1707 |
| ENSG00000125651 | 0.2752 | 0.0050 | 0.0343 |
| ENSG00000161914 | 0.2752 | 0.0002 | 0.0026 |
| ENSG00000083844 | 0.2750 | 0.0189 | 0.0918 |
| ENSG00000172888 | 0.2737 | 0.0220 | 0.1027 |
| ENSG00000163214 | 0.2735 | 0.0090 | 0.0531 |
| ENSG00000178229 | 0.2728 | 0.0319 | 0.1333 |
| ENSG00000198131 | 0.2718 | 0.0371 | 0.1485 |
| ENSG00000152443 | 0.2714 | 0.0052 | 0.0357 |
| ENSG00000104903 | 0.2713 | 0.2144 | 0.4619 |
| ENSG00000083642 | 0.2695 | 0.0089 | 0.0527 |
| ENSG00000075407 | 0.2681 | 0.0292 | 0.1254 |
| ENSG00000186660 | 0.2680 | 0.0144 | 0.0752 |
| ENSG00000105698 | 0.2659 | 0.0493 | 0.1816 |
| ENSG00000169635 | 0.2651 | 0.0066 | 0.0425 |
| ENSG00000089902 | 0.2647 | 0.1132 | 0.3161 |
| ENSG00000188994 | 0.2632 | 0.0700 | 0.2311 |
| ENSG00000066422 | 0.2594 | 0.0187 | 0.0913 |
| ENSG00000189079 | 0.2594 | 0.0488 | 0.1803 |
| ENSG00000197063 | 0.2593 | 0.0162 | 0.0822 |
| ENSG00000115020 | 0.2591 | 0.0393 | 0.1549 |
| ENSG00000178764 | 0.2590 | 0.0605 | 0.2091 |
| ENSG00000118260 | 0.2589 | 0.0102 | 0.0584 |
| ENSG00000170325 | 0.2580 | 0.0211 | 0.0997 |
| ENSG00000196922 | 0.2573 | 0.0079 | 0.0486 |
| ENSG00000144026 | 0.2546 | 0.0458 | 0.1722 |
| ENSG00000177873 | 0.2534 | 0.0005 | 0.0058 |
| ENSG00000072121 | 0.2530 | 0.0283 | 0.1225 |
| ENSG00000131116 | 0.2523 | 0.1213 | 0.3312 |
| ENSG00000198783 | 0.2518 | 0.0063 | 0.0411 |
| ENSG00000106459 | 0.2515 | 0.0014 | 0.0132 |
| ENSG00000154832 | 0.2500 | 0.0484 | 0.1792 |
| ENSG00000065029 | 0.2482 | 0.0169 | 0.0846 |
| ENSG00000198182 | 0.2476 | 0.0454 | 0.1713 |
| ENSG00000120075 | 0.2459 | 0.3866 | 0.6249 |
| ENSG00000141503 | 0.2458 | 0.1822 | 0.4189 |
| ENSG00000122877 | 0.2446 | 0.3215 | 0.5625 |
| ENSG00000138709 | 0.2445 | 0.0151 | 0.0780 |
| ENSG00000125846 | 0.2442 | 0.0165 | 0.0832 |
| ENSG00000168813 | 0.2439 | 0.0278 | 0.1212 |
| ENSG00000197302 | 0.2439 | 0.0011 | 0.0104 |
| ENSG00000169249 | 0.2435 | 0.0988 | 0.2897 |
| ENSG00000175727 | 0.2433 | 0.2461 | 0.5024 |
| ENSG00000102908 | 0.2431 | 0.2034 | 0.4479 |
| ENSG00000135148 | 0.2429 | 0.0856 | 0.2632 |
| ENSG00000073584 | 0.2420 | 0.0987 | 0.2894 |
| ENSG00000196214 | 0.2412 | 0.0018 | 0.0156 |
| ENSG00000198482 | 0.2407 | 0.0648 | 0.2193 |
| ENSG00000150347 | 0.2402 | 0.1806 | 0.4170 |
| ENSG00000151612 | 0.2382 | 0.1253 | 0.3379 |
| ENSG00000197933 | 0.2378 | 0.0899 | 0.2720 |
| ENSG00000084112 | 0.2372 | 0.1058 | 0.3027 |
| ENSG00000204859 | 0.2371 | 0.0156 | 0.0799 |
| ENSG00000197020 | 0.2365 | 0.0078 | 0.0478 |
| ENSG00000172830 | 0.2357 | 0.1736 | 0.4074 |
| ENSG00000167384 | 0.2353 | 0.0077 | 0.0476 |
| ENSG00000084093 | 0.2337 | 0.0481 | 0.1784 |
| ENSG00000204524 | 0.2332 | 0.0230 | 0.1059 |
| ENSG00000101544 | 0.2328 | 0.0146 | 0.0761 |
| ENSG00000176946 | 0.2324 | 0.0448 | 0.1695 |
| ENSG00000160908 | 0.2323 | 0.0125 | 0.0680 |
| ENSG00000181666 | 0.2321 | 0.0349 | 0.1422 |
| ENSG00000198265 | 0.2311 | 0.0455 | 0.1714 |
| ENSG00000143614 | 0.2311 | 0.0686 | 0.2282 |
| ENSG00000028277 | 0.2305 | 0.3331 | 0.5707 |
| ENSG00000120837 | 0.2302 | 0.0602 | 0.2084 |
| ENSG00000166147 | 0.2299 | 0.4267 | 0.6635 |
| ENSG00000169131 | 0.2296 | 0.1379 | 0.3590 |
| ENSG00000100150 | 0.2293 | 0.0110 | 0.0617 |
| ENSG00000131196 | 0.2293 | 0.1891 | 0.4286 |
| ENSG00000164011 | 0.2286 | 0.0534 | 0.1916 |
| ENSG00000203883 | 0.2281 | 0.2644 | 0.5244 |
| ENSG00000177700 | 0.2281 | 0.2070 | 0.4522 |
| ENSG00000139651 | 0.2280 | 0.0535 | 0.1918 |
| ENSG00000187626 | 0.2276 | 0.0138 | 0.0730 |
| ENSG00000184677 | 0.2271 | 0.0965 | 0.2849 |
| ENSG00000099326 | 0.2252 | 0.0464 | 0.1738 |
| ENSG00000177842 | 0.2251 | 0.0563 | 0.1987 |
| ENSG00000175691 | 0.2241 | 0.0070 | 0.0443 |
| ENSG00000189042 | 0.2241 | 0.0169 | 0.0848 |
| ENSG00000115966 | 0.2224 | 0.0498 | 0.1829 |
| ENSG00000160113 | 0.2219 | 0.3180 | 0.5625 |
| ENSG00000102901 | 0.2211 | 0.0359 | 0.1451 |
| ENSG00000087510 | 0.2209 | 0.5440 | 0.7675 |
| ENSG00000106052 | 0.2184 | 0.0648 | 0.2193 |
| ENSG00000116990 | 0.2182 | 0.3238 | 0.5636 |
| ENSG00000173480 | 0.2172 | 0.0278 | 0.1211 |
| ENSG00000068323 | 0.2160 | 0.1058 | 0.3027 |
| ENSG00000130382 | 0.2159 | 0.1330 | 0.3513 |
| ENSG00000172292 | 0.2151 | 0.1521 | 0.3814 |
| ENSG00000177125 | 0.2150 | 0.0211 | 0.0995 |
| ENSG00000105879 | 0.2148 | 0.0622 | 0.2132 |
| ENSG00000123200 | 0.2127 | 0.2283 | 0.4799 |
| ENSG00000162601 | 0.2115 | 0.1771 | 0.4121 |
| ENSG00000103510 | 0.2109 | 0.0750 | 0.2423 |
| ENSG00000083223 | 0.2109 | 0.1979 | 0.4408 |
| ENSG00000072736 | 0.2066 | 0.1039 | 0.2991 |
| ENSG00000075292 | 0.2015 | 0.1231 | 0.3343 |
| ENSG00000198815 | 0.2015 | 0.0638 | 0.2172 |
| ENSG00000038219 | 0.2010 | 0.1680 | 0.4004 |
| ENSG00000170365 | 0.2004 | 0.0769 | 0.2460 |
| ENSG00000091009 | 0.2002 | 0.0090 | 0.0532 |
| ENSG00000156374 | 0.1998 | 0.0859 | 0.2639 |
| ENSG00000101665 | 0.1969 | 0.2917 | 0.5570 |
| ENSG00000007545 | 0.1964 | 0.0441 | 0.1676 |
| ENSG00000089775 | 0.1960 | 0.0433 | 0.1658 |
| ENSG00000160685 | 0.1956 | 0.4140 | 0.6515 |
| ENSG00000125845 | 0.1948 | 0.4995 | 0.7305 |
| ENSG00000153560 | 0.1941 | 0.0807 | 0.2534 |
| ENSG00000125520 | 0.1903 | 0.3833 | 0.6211 |
| ENSG00000129028 | 0.1898 | 0.1414 | 0.3651 |
| ENSG00000172379 | 0.1895 | 0.4694 | 0.7029 |
| ENSG00000162714 | 0.1895 | 0.2088 | 0.4543 |
| ENSG00000086102 | 0.1884 | 0.0790 | 0.2501 |
| ENSG00000110851 | 0.1879 | 0.1461 | 0.3729 |
| ENSG00000130856 | 0.1872 | 0.0679 | 0.2266 |
| ENSG00000170631 | 0.1868 | 0.0114 | 0.0635 |
| ENSG00000137185 | 0.1868 | 0.0250 | 0.1125 |
| ENSG00000167232 | 0.1861 | 0.1111 | 0.3123 |
| ENSG00000122299 | 0.1850 | 0.1781 | 0.4136 |
| ENSG00000059145 | 0.1846 | 0.0633 | 0.2158 |
| ENSG00000109381 | 0.1843 | 0.1389 | 0.3609 |
| ENSG00000064961 | 0.1829 | 0.0742 | 0.2406 |
| ENSG00000135164 | 0.1828 | 0.2309 | 0.4830 |
| ENSG00000168310 | 0.1827 | 0.1335 | 0.3520 |
| ENSG00000174720 | 0.1813 | 0.1324 | 0.3503 |
| ENSG00000185219 | 0.1809 | 0.1746 | 0.4086 |
| ENSG00000124444 | 0.1807 | 0.0760 | 0.2445 |
| ENSG00000177463 | 0.1804 | 0.2130 | 0.4601 |
| ENSG00000105419 | 0.1800 | 0.1609 | 0.3913 |
| ENSG00000187815 | 0.1797 | 0.0318 | 0.1331 |
| ENSG00000103343 | 0.1793 | 0.0926 | 0.2771 |
| ENSG00000171443 | 0.1779 | 0.1963 | 0.4386 |
| ENSG00000118058 | 0.1775 | 0.2687 | 0.5297 |
| ENSG00000174652 | 0.1775 | 0.2151 | 0.4630 |
| ENSG00000145734 | 0.1769 | 0.1932 | 0.4342 |
| ENSG00000105939 | 0.1769 | 0.0807 | 0.2534 |
| ENSG00000198551 | 0.1767 | 0.0899 | 0.2721 |
| ENSG00000154957 | 0.1763 | 0.0275 | 0.1204 |
| ENSG00000186448 | 0.1746 | 0.0547 | 0.1948 |
| ENSG00000166529 | 0.1726 | 0.1701 | 0.4029 |
| ENSG00000103199 | 0.1706 | 0.0426 | 0.1640 |
| ENSG00000204304 | 0.1689 | 0.2320 | 0.4844 |
| ENSG00000176407 | 0.1682 | 0.0481 | 0.1783 |
| ENSG00000117505 | 0.1680 | 0.0737 | 0.2396 |
| ENSG00000139154 | 0.1639 | 0.1867 | 0.4252 |
| ENSG00000197024 | 0.1632 | 0.1409 | 0.3641 |
| ENSG00000198740 | 0.1614 | 0.1876 | 0.4267 |
| ENSG00000140479 | 0.1596 | 0.2918 | 0.5571 |
| ENSG00000160007 | 0.1595 | 0.2794 | 0.5425 |
| ENSG00000197363 | 0.1594 | 0.1899 | 0.4298 |
| ENSG00000004534 | 0.1581 | 0.2795 | 0.5426 |
| ENSG00000178951 | 0.1566 | 0.3363 | 0.5741 |
| ENSG00000159263 | 0.1558 | 0.5854 | 0.7999 |
| ENSG00000167182 | 0.1551 | 0.1379 | 0.3591 |
| ENSG00000131115 | 0.1546 | 0.0899 | 0.2721 |
| ENSG00000116016 | 0.1537 | 0.2723 | 0.5340 |
| ENSG00000170260 | 0.1511 | 0.1957 | 0.4377 |
| ENSG00000036257 | 0.1491 | 0.1665 | 0.3982 |
| ENSG00000157557 | 0.1488 | 0.5009 | 0.7317 |
| ENSG00000078140 | 0.1486 | 0.1200 | 0.3288 |
| ENSG00000048405 | 0.1484 | 0.1474 | 0.3750 |
| ENSG00000141002 | 0.1476 | 0.1234 | 0.3347 |
| ENSG00000163848 | 0.1461 | 0.1516 | 0.3811 |
| ENSG00000128000 | 0.1441 | 0.1931 | 0.4340 |
| ENSG00000172273 | 0.1434 | 0.0503 | 0.1841 |
| ENSG00000178935 | 0.1433 | 0.2724 | 0.5342 |
| ENSG00000083812 | 0.1427 | 0.0700 | 0.2312 |
| ENSG00000041988 | 0.1417 | 0.1355 | 0.3549 |
| ENSG00000112033 | 0.1393 | 0.4321 | 0.6688 |
| ENSG00000198081 | 0.1391 | 0.1731 | 0.4067 |
| ENSG00000196456 | 0.1379 | 0.4139 | 0.6513 |
| ENSG00000140396 | 0.1353 | 0.3725 | 0.6104 |
| ENSG00000163635 | 0.1340 | 0.1966 | 0.4389 |
| ENSG00000161642 | 0.1337 | 0.6146 | 0.8224 |
| ENSG00000090612 | 0.1333 | 0.1541 | 0.3831 |
| ENSG00000130544 | 0.1331 | 0.0753 | 0.2429 |
| ENSG00000133884 | 0.1319 | 0.2568 | 0.5153 |
| ENSG00000167380 | 0.1316 | 0.1532 | 0.3821 |
| ENSG00000173275 | 0.1311 | 0.2420 | 0.4973 |
| ENSG00000112561 | 0.1310 | 0.4272 | 0.6640 |
| ENSG00000172819 | 0.1305 | 0.6830 | 0.8737 |
| ENSG00000164048 | 0.1297 | 0.2992 | 0.5625 |
| ENSG00000123411 | 0.1297 | 0.2865 | 0.5508 |
| ENSG00000167785 | 0.1286 | 0.1983 | 0.4411 |
| ENSG00000196267 | 0.1272 | 0.1144 | 0.3185 |
| ENSG00000147601 | 0.1272 | 0.1720 | 0.4054 |
| ENSG00000167528 | 0.1270 | 0.2559 | 0.5141 |
| ENSG00000132478 | 0.1268 | 0.2244 | 0.4748 |
| ENSG00000166888 | 0.1264 | 0.3156 | 0.5625 |
| ENSG00000182318 | 0.1245 | 0.0596 | 0.2070 |
| ENSG00000198040 | 0.1242 | 0.3919 | 0.6302 |
| ENSG00000196152 | 0.1238 | 0.1211 | 0.3309 |
| ENSG00000081665 | 0.1237 | 0.2311 | 0.4831 |
| ENSG00000082641 | 0.1235 | 0.4175 | 0.6549 |
| ENSG00000185669 | 0.1234 | 0.4775 | 0.7102 |
| ENSG00000081386 | 0.1222 | 0.2405 | 0.4952 |
| ENSG00000186017 | 0.1214 | 0.2386 | 0.4929 |
| ENSG00000127483 | 0.1212 | 0.4397 | 0.6760 |
| ENSG00000004975 | 0.1205 | 0.3589 | 0.5966 |
| ENSG00000189060 | 0.1198 | 0.5023 | 0.7328 |
| ENSG00000204103 | 0.1194 | 0.5484 | 0.7712 |
| ENSG00000171425 | 0.1192 | 0.3770 | 0.6150 |
| ENSG00000188070 | 0.1172 | 0.5371 | 0.7620 |
| ENSG00000066827 | 0.1138 | 0.1122 | 0.3142 |
| ENSG00000188786 | 0.1136 | 0.3938 | 0.6323 |
| ENSG00000147421 | 0.1132 | 0.3411 | 0.5788 |
| ENSG00000010818 | 0.1125 | 0.4855 | 0.7176 |
| ENSG00000166266 | 0.1107 | 0.2702 | 0.5316 |
| ENSG00000198315 | 0.1106 | 0.5171 | 0.7451 |
| ENSG00000095574 | 0.1099 | 0.3001 | 0.5625 |
| ENSG00000134744 | 0.1088 | 0.4012 | 0.6393 |
| ENSG00000168795 | 0.1086 | 0.5173 | 0.7454 |
| ENSG00000133606 | 0.1075 | 0.1846 | 0.4224 |
| ENSG00000167766 | 0.1057 | 0.5159 | 0.7444 |
| ENSG00000179922 | 0.1051 | 0.3556 | 0.5934 |
| ENSG00000168826 | 0.1031 | 0.2024 | 0.4464 |
| ENSG00000215021 | 0.1025 | 0.4820 | 0.7146 |
| ENSG00000166454 | 0.1018 | 0.3327 | 0.5705 |
| ENSG00000168916 | 0.1013 | 0.4508 | 0.6861 |
| ENSG00000166478 | 0.0998 | 0.1302 | 0.3465 |
| ENSG00000117000 | 0.0994 | 0.4468 | 0.6825 |
| ENSG00000171574 | 0.0986 | 0.2859 | 0.5501 |
| ENSG00000055130 | 0.0977 | 0.3697 | 0.6077 |
| ENSG00000196646 | 0.0975 | 0.2387 | 0.4930 |
| ENSG00000170653 | 0.0975 | 0.4877 | 0.7201 |
| ENSG00000151702 | 0.0966 | 0.5555 | 0.7768 |
| ENSG00000089335 | 0.0958 | 0.5160 | 0.7445 |
| ENSG00000134046 | 0.0939 | 0.4123 | 0.6496 |
| ENSG00000121486 | 0.0935 | 0.2758 | 0.5383 |
| ENSG00000141027 | 0.0932 | 0.5628 | 0.7827 |
| ENSG00000205189 | 0.0924 | 0.6353 | 0.8377 |
| ENSG00000135547 | 0.0899 | 0.4469 | 0.6825 |
| ENSG00000132846 | 0.0897 | 0.5725 | 0.7901 |
| ENSG00000169155 | 0.0888 | 0.4700 | 0.7034 |
| ENSG00000100722 | 0.0870 | 0.2272 | 0.4786 |
| ENSG00000070444 | 0.0857 | 0.4168 | 0.6542 |
| ENSG00000115112 | 0.0855 | 0.8005 | 0.9530 |
| ENSG00000178028 | 0.0850 | 0.2534 | 0.5112 |
| ENSG00000083838 | 0.0844 | 0.2384 | 0.4926 |
| ENSG00000196387 | 0.0838 | 0.3994 | 0.6377 |
| ENSG00000116539 | 0.0836 | 0.5995 | 0.8110 |
| ENSG00000103994 | 0.0829 | 0.5689 | 0.7872 |
| ENSG00000177030 | 0.0818 | 0.4028 | 0.6409 |
| ENSG00000136169 | 0.0816 | 0.4222 | 0.6592 |
| ENSG00000162086 | 0.0807 | 0.4367 | 0.6732 |
| ENSG00000168286 | 0.0807 | 0.4432 | 0.6793 |
| ENSG00000115350 | 0.0796 | 0.5997 | 0.8112 |
| ENSG00000198146 | 0.0784 | 0.6812 | 0.8722 |
| ENSG00000121864 | 0.0769 | 0.4802 | 0.7130 |
| ENSG00000074657 | 0.0768 | 0.7243 | 0.9018 |
| ENSG00000174796 | 0.0761 | 0.3937 | 0.6323 |
| ENSG00000169955 | 0.0742 | 0.4567 | 0.6913 |
| ENSG00000131931 | 0.0732 | 0.4243 | 0.6610 |
| ENSG00000171448 | 0.0730 | 0.6180 | 0.8250 |
| ENSG00000131910 | 0.0728 | 0.9030 | 1.0000 |
| ENSG00000186951 | 0.0724 | 0.6689 | 0.8632 |
| ENSG00000196150 | 0.0722 | 0.3338 | 0.5715 |
| ENSG00000177683 | 0.0713 | 0.5295 | 0.7556 |
| ENSG00000014824 | 0.0706 | 0.3999 | 0.6382 |
| ENSG00000113658 | 0.0701 | 0.6310 | 0.8343 |
| ENSG00000152475 | 0.0681 | 0.5748 | 0.7918 |
| ENSG00000005889 | 0.0678 | 0.6550 | 0.8524 |
| ENSG00000184436 | 0.0670 | 0.6236 | 0.8292 |
| ENSG00000154727 | 0.0662 | 0.5577 | 0.7785 |
| ENSG00000172667 | 0.0656 | 0.6362 | 0.8384 |
| ENSG00000107223 | 0.0647 | 0.6839 | 0.8743 |
| ENSG00000120093 | 0.0641 | 0.7703 | 0.9328 |
| ENSG00000085274 | 0.0630 | 0.4863 | 0.7185 |
| ENSG00000177374 | 0.0630 | 0.7642 | 0.9288 |
| ENSG00000118620 | 0.0610 | 0.6447 | 0.8446 |
| ENSG00000167034 | 0.0603 | 0.8300 | 0.9719 |
| ENSG00000134852 | 0.0581 | 0.6231 | 0.8289 |
| ENSG00000074047 | 0.0577 | 0.7407 | 0.9130 |
| ENSG00000143437 | 0.0548 | 0.5641 | 0.7836 |
| ENSG00000178338 | 0.0542 | 0.6574 | 0.8543 |
| ENSG00000107175 | 0.0542 | 0.7332 | 0.9082 |
| ENSG00000182141 | 0.0539 | 0.5855 | 0.8000 |
| ENSG00000157554 | 0.0523 | 0.6302 | 0.8337 |
| ENSG00000133794 | 0.0516 | 0.6917 | 0.8795 |
| ENSG00000111269 | 0.0516 | 0.6442 | 0.8443 |
| ENSG00000140836 | 0.0514 | 0.8013 | 0.9536 |
| ENSG00000057935 | 0.0513 | 0.5533 | 0.7751 |
| ENSG00000185670 | 0.0505 | 0.4994 | 0.7304 |
| ENSG00000166848 | 0.0484 | 0.6417 | 0.8425 |
| ENSG00000204231 | 0.0477 | 0.6275 | 0.8318 |
| ENSG00000173875 | 0.0432 | 0.6562 | 0.8533 |
| ENSG00000170430 | 0.0421 | 0.8111 | 0.9597 |
| ENSG00000184517 | 0.0415 | 0.7201 | 0.8992 |
| ENSG00000160094 | 0.0402 | 0.7975 | 0.9510 |
| ENSG00000083828 | 0.0395 | 0.6442 | 0.8442 |
| ENSG00000136504 | 0.0394 | 0.7518 | 0.9206 |
| ENSG00000185551 | 0.0379 | 0.8885 | 1.0000 |
| ENSG00000136997 | 0.0374 | 0.8746 | 0.9997 |
| ENSG00000172466 | 0.0366 | 0.7353 | 0.9094 |
| ENSG00000188785 | 0.0364 | 0.7614 | 0.9269 |
| ENSG00000135870 | 0.0353 | 0.7798 | 0.9391 |
| ENSG00000156273 | 0.0345 | 0.8194 | 0.9649 |
| ENSG00000058729 | 0.0340 | 0.6419 | 0.8425 |
| ENSG00000117036 | 0.0330 | 0.7756 | 0.9362 |
| ENSG00000186812 | 0.0320 | 0.7777 | 0.9376 |
| ENSG00000177352 | 0.0291 | 0.7876 | 0.9444 |
| ENSG00000177311 | 0.0287 | 0.8510 | 0.9847 |
| ENSG00000101695 | 0.0287 | 0.8692 | 0.9963 |
| ENSG00000163939 | 0.0275 | 0.8402 | 0.9786 |
| ENSG00000197857 | 0.0271 | 0.7409 | 0.9131 |
| ENSG00000141644 | 0.0270 | 0.7504 | 0.9195 |
| ENSG00000142611 | 0.0256 | 0.9068 | 1.0000 |
| ENSG00000100319 | 0.0254 | 0.8418 | 0.9792 |
| ENSG00000184939 | 0.0243 | 0.8423 | 0.9795 |
| ENSG00000124191 | 0.0221 | 0.9128 | 1.0000 |
| ENSG00000186272 | 0.0210 | 0.7708 | 0.9330 |
| ENSG00000075975 | 0.0189 | 0.8464 | 0.9821 |
| ENSG00000204060 | 0.0186 | 0.9254 | 1.0000 |
| ENSG00000083817 | 0.0175 | 0.8642 | 0.9931 |
| ENSG00000187792 | 0.0171 | 0.8766 | 1.0000 |
| ENSG00000198839 | 0.0169 | 0.8483 | 0.9831 |
| ENSG00000125945 | 0.0166 | 0.9273 | 1.0000 |
| ENSG00000215421 | 0.0166 | 0.8237 | 0.9676 |
| ENSG00000198160 | 0.0161 | 0.8614 | 0.9912 |
| ENSG00000134317 | 0.0150 | 0.9689 | 1.0000 |
| ENSG00000105708 | 0.0135 | 0.9097 | 1.0000 |
| ENSG00000170949 | 0.0133 | 0.9196 | 1.0000 |
| ENSG00000160199 | 0.0131 | 0.8944 | 1.0000 |
| ENSG00000003756 | 0.0129 | 0.9225 | 1.0000 |
| ENSG00000144369 | 0.0124 | 0.9325 | 1.0000 |
| ENSG00000169057 | 0.0124 | 0.9307 | 1.0000 |
| ENSG00000179965 | 0.0070 | 0.9511 | 1.0000 |
| ENSG00000176472 | 0.0041 | 0.9549 | 1.0000 |
| ENSG00000114439 | 0.0023 | 0.9886 | 1.0000 |
| ENSG00000173011 | 0.0022 | 0.9833 | 1.0000 |
| ENSG00000175387 | 0.0009 | 0.9890 | 1.0000 |
| ENSG00000123636 | 0.0009 | 0.9939 | 1.0000 |
| ENSG00000149922 | 0.0004 | 0.9979 | 1.0000 |
| ENSG00000198911 | 0.0004 | 0.9978 | 1.0000 |
| ENSG00000178096 | -0.0001 | 0.9993 | 1.0000 |
| ENSG00000183621 | -0.0023 | 0.9849 | 1.0000 |
| ENSG00000174738 | -0.0024 | 0.9881 | 1.0000 |
| ENSG00000125952 | -0.0030 | 0.9757 | 1.0000 |
| ENSG00000126351 | -0.0037 | 0.9872 | 1.0000 |
| ENSG00000186020 | -0.0044 | 0.9695 | 1.0000 |
| ENSG00000186814 | -0.0045 | 0.9610 | 1.0000 |
| ENSG00000171469 | -0.0057 | 0.9631 | 1.0000 |
| ENSG00000196705 | -0.0126 | 0.9447 | 1.0000 |
| ENSG00000161277 | -0.0129 | 0.9357 | 1.0000 |
| ENSG00000060138 | -0.0162 | 0.9407 | 1.0000 |
| ENSG00000142556 | -0.0172 | 0.8810 | 1.0000 |
| ENSG00000142599 | -0.0173 | 0.9236 | 1.0000 |
| ENSG00000074219 | -0.0202 | 0.9376 | 1.0000 |
| ENSG00000057657 | -0.0214 | 0.9473 | 1.0000 |
| ENSG00000146007 | -0.0223 | 0.7845 | 0.9422 |
| ENSG00000118689 | -0.0338 | 0.7932 | 0.9482 |
| ENSG00000131845 | -0.0350 | 0.7951 | 0.9496 |
| ENSG00000176542 | -0.0366 | 0.7495 | 0.9190 |
| ENSG00000055609 | -0.0402 | 0.7810 | 0.9398 |
| ENSG00000155545 | -0.0423 | 0.7512 | 0.9202 |
| ENSG00000030419 | -0.0438 | 0.8671 | 0.9950 |
| ENSG00000076356 | -0.0444 | 0.7934 | 0.9483 |
| ENSG00000150907 | -0.0448 | 0.7660 | 0.9300 |
| ENSG00000122691 | -0.0474 | 0.8276 | 0.9704 |
| ENSG00000148143 | -0.0483 | 0.8010 | 0.9533 |
| ENSG00000140632 | -0.0490 | 0.6569 | 0.8539 |
| ENSG00000160062 | -0.0501 | 0.6409 | 0.8418 |
| ENSG00000105997 | -0.0533 | 0.8116 | 0.9600 |
| ENSG00000196189 | -0.0557 | 0.8289 | 0.9712 |
| ENSG00000127603 | -0.0557 | 0.7946 | 0.9492 |
| ENSG00000133250 | -0.0569 | 0.5901 | 0.8035 |
| ENSG00000105497 | -0.0574 | 0.4602 | 0.6946 |
| ENSG00000182463 | -0.0586 | 0.7349 | 0.9092 |
| ENSG00000197050 | -0.0601 | 0.5927 | 0.8057 |
| ENSG00000025156 | -0.0602 | 0.7179 | 0.8981 |
| ENSG00000109787 | -0.0617 | 0.7113 | 0.8937 |
| ENSG00000213096 | -0.0637 | 0.5709 | 0.7887 |
| ENSG00000114861 | -0.0639 | 0.6832 | 0.8738 |
| ENSG00000189298 | -0.0648 | 0.4924 | 0.7245 |
| ENSG00000137265 | -0.0657 | 0.8390 | 0.9779 |
| ENSG00000095794 | -0.0658 | 0.6785 | 0.8702 |
| ENSG00000121417 | -0.0667 | 0.5774 | 0.7936 |
| ENSG00000175105 | -0.0713 | 0.5786 | 0.7945 |
| ENSG00000115738 | -0.0713 | 0.7991 | 0.9521 |
| ENSG00000131408 | -0.0723 | 0.5758 | 0.7925 |
| ENSG00000196628 | -0.0726 | 0.6061 | 0.8163 |
| ENSG00000102189 | -0.0745 | 0.6398 | 0.8412 |
| ENSG00000123933 | -0.0782 | 0.5438 | 0.7673 |
| ENSG00000141030 | -0.0805 | 0.4451 | 0.6810 |
| ENSG00000188283 | -0.0862 | 0.3706 | 0.6086 |
| ENSG00000173276 | -0.0911 | 0.4275 | 0.6644 |
| ENSG00000177200 | -0.1011 | 0.4150 | 0.6524 |
| ENSG00000198105 | -0.1018 | 0.4457 | 0.6814 |
| ENSG00000169740 | -0.1044 | 0.5583 | 0.7789 |
| ENSG00000196653 | -0.1045 | 0.3612 | 0.5989 |
| ENSG00000019549 | -0.1052 | 0.5123 | 0.7413 |
| ENSG00000179348 | -0.1052 | 0.4479 | 0.6834 |
| ENSG00000141646 | -0.1062 | 0.2735 | 0.5355 |
| ENSG00000116731 | -0.1065 | 0.3885 | 0.6266 |
| ENSG00000180667 | -0.1067 | 0.7279 | 0.9044 |
| ENSG00000171606 | -0.1083 | 0.1965 | 0.4387 |
| ENSG00000166200 | -0.1106 | 0.3411 | 0.5787 |
| ENSG00000113761 | -0.1120 | 0.2209 | 0.4701 |
| ENSG00000177045 | -0.1121 | 0.5391 | 0.7634 |
| ENSG00000184481 | -0.1131 | 0.4423 | 0.6785 |
| ENSG00000197841 | -0.1170 | 0.2412 | 0.4962 |
| ENSG00000137871 | -0.1183 | 0.3272 | 0.5659 |
| ENSG00000065970 | -0.1184 | 0.4448 | 0.6806 |
| ENSG00000117625 | -0.1191 | 0.3688 | 0.6067 |
| ENSG00000161265 | -0.1225 | 0.3705 | 0.6085 |
| ENSG00000006468 | -0.1251 | 0.5160 | 0.7445 |
| ENSG00000173757 | -0.1270 | 0.4695 | 0.7030 |
| ENSG00000198795 | -0.1352 | 0.3110 | 0.5625 |
| ENSG00000173917 | -0.1406 | 0.5950 | 0.8076 |
| ENSG00000136451 | -0.1425 | 0.3252 | 0.5644 |
| ENSG00000172977 | -0.1426 | 0.1153 | 0.3203 |
| ENSG00000196605 | -0.1436 | 0.2297 | 0.4815 |
| ENSG00000060566 | -0.1451 | 0.8294 | 0.9715 |
| ENSG00000108509 | -0.1459 | 0.2435 | 0.4992 |
| ENSG00000152454 | -0.1485 | 0.2455 | 0.5017 |
| ENSG00000165655 | -0.1492 | 0.5028 | 0.7331 |
| ENSG00000105516 | -0.1526 | 0.3502 | 0.5883 |
| ENSG00000180535 | -0.1536 | 0.7180 | 0.8982 |
| ENSG00000079337 | -0.1538 | 0.2892 | 0.5540 |
| ENSG00000171735 | -0.1572 | 0.1176 | 0.3244 |
| ENSG00000118922 | -0.1583 | 0.3636 | 0.6013 |
| ENSG00000119866 | -0.1601 | 0.4131 | 0.6505 |
| ENSG00000176842 | -0.1615 | 0.5009 | 0.7317 |
| ENSG00000092203 | -0.1656 | 0.0416 | 0.1613 |
| ENSG00000198521 | -0.1678 | 0.1885 | 0.4279 |
| ENSG00000102302 | -0.1691 | 0.3562 | 0.5940 |
| ENSG00000140386 | -0.1714 | 0.0764 | 0.2450 |
| ENSG00000181722 | -0.1718 | 0.2907 | 0.5557 |
| ENSG00000067082 | -0.1749 | 0.2549 | 0.5130 |
| ENSG00000110171 | -0.1766 | 0.1212 | 0.3309 |
| ENSG00000182742 | -0.1766 | 0.3373 | 0.5751 |
| ENSG00000118263 | -0.1835 | 0.2553 | 0.5133 |
| ENSG00000188321 | -0.1847 | 0.1366 | 0.3567 |
| ENSG00000128272 | -0.1875 | 0.1924 | 0.4335 |
| ENSG00000197013 | -0.1877 | 0.1049 | 0.3010 |
| ENSG00000116604 | -0.1883 | 0.2792 | 0.5423 |
| ENSG00000196700 | -0.1951 | 0.3636 | 0.6013 |
| ENSG00000138663 | -0.1951 | 0.0500 | 0.1834 |
| ENSG00000171970 | -0.1954 | 0.2262 | 0.4773 |
| ENSG00000186918 | -0.1956 | 0.2025 | 0.4466 |
| ENSG00000213015 | -0.1961 | 0.2610 | 0.5204 |
| ENSG00000171843 | -0.2080 | 0.0453 | 0.1711 |
| ENSG00000139793 | -0.2092 | 0.2137 | 0.4608 |
| ENSG00000147862 | -0.2100 | 0.3623 | 0.5999 |
| ENSG00000215271 | -0.2137 | 0.0182 | 0.0894 |
| ENSG00000165156 | -0.2138 | 0.1332 | 0.3516 |
| ENSG00000119919 | -0.2146 | 0.2811 | 0.5448 |
| ENSG00000197619 | -0.2173 | 0.0713 | 0.2340 |
| ENSG00000171056 | -0.2177 | 0.3898 | 0.6280 |
| ENSG00000153234 | -0.2181 | 0.5412 | 0.7650 |
| ENSG00000083814 | -0.2204 | 0.0806 | 0.2532 |
| ENSG00000072364 | -0.2222 | 0.1813 | 0.4178 |
| ENSG00000156030 | -0.2235 | 0.1045 | 0.3003 |
| ENSG00000005102 | -0.2250 | 0.2237 | 0.4737 |
| ENSG00000180964 | -0.2268 | 0.2149 | 0.4626 |
| ENSG00000043039 | -0.2293 | 0.5786 | 0.7945 |
| ENSG00000113595 | -0.2309 | 0.0455 | 0.1714 |
| ENSG00000169554 | -0.2317 | 0.1182 | 0.3257 |
| ENSG00000186350 | -0.2330 | 0.1276 | 0.3419 |
| ENSG00000182158 | -0.2344 | 0.1318 | 0.3492 |
| ENSG00000135365 | -0.2346 | 0.0959 | 0.2838 |
| ENSG00000126705 | -0.2478 | 0.3192 | 0.5625 |
| ENSG00000164330 | -0.2505 | 0.2479 | 0.5044 |
| ENSG00000181444 | -0.2533 | 0.2089 | 0.4545 |
| ENSG00000155792 | -0.2544 | 0.2623 | 0.5219 |
| ENSG00000136936 | -0.2608 | 0.0280 | 0.1216 |
| ENSG00000143365 | -0.2626 | 0.3540 | 0.5919 |
| ENSG00000185022 | -0.2656 | 0.2510 | 0.5082 |
| ENSG00000196378 | -0.2690 | 0.0627 | 0.2144 |
| ENSG00000081189 | -0.2750 | 0.1784 | 0.4140 |
| ENSG00000172059 | -0.2793 | 0.1723 | 0.4057 |
| ENSG00000180787 | -0.2794 | 0.0377 | 0.1501 |
| ENSG00000084676 | -0.2807 | 0.0326 | 0.1354 |
| ENSG00000130940 | -0.2851 | 0.0572 | 0.2010 |
| ENSG00000178982 | -0.2935 | 0.0452 | 0.1707 |
| ENSG00000131381 | -0.2941 | 0.0633 | 0.2159 |
| ENSG00000104447 | -0.2944 | 0.2129 | 0.4600 |
| ENSG00000152601 | -0.2948 | 0.0876 | 0.2674 |
| ENSG00000181894 | -0.2952 | 0.0174 | 0.0867 |
| ENSG00000197343 | -0.2953 | 0.0825 | 0.2568 |
| ENSG00000153814 | -0.2971 | 0.1478 | 0.3755 |
| ENSG00000188177 | -0.2989 | 0.0422 | 0.1627 |
| ENSG00000171476 | -0.3069 | 0.4690 | 0.7025 |
| ENSG00000197951 | -0.3078 | 0.0154 | 0.0793 |
| ENSG00000114126 | -0.3154 | 0.1122 | 0.3143 |
| ENSG00000068305 | -0.3165 | 0.0866 | 0.2652 |
| ENSG00000196428 | -0.3213 | 0.0418 | 0.1617 |
| ENSG00000165804 | -0.3214 | 0.0661 | 0.2221 |
| ENSG00000197062 | -0.3231 | 0.0253 | 0.1134 |
| ENSG00000198093 | -0.3256 | 0.0069 | 0.0437 |
| ENSG00000198353 | -0.3315 | 0.1068 | 0.3047 |
| ENSG00000165030 | -0.3316 | 0.0615 | 0.2118 |
| ENSG00000164684 | -0.3359 | 0.0534 | 0.1917 |
| ENSG00000130818 | -0.3370 | 0.0685 | 0.2279 |
| ENSG00000102804 | -0.3385 | 0.0535 | 0.1919 |
| ENSG00000164463 | -0.3479 | 0.0079 | 0.0485 |
| ENSG00000077092 | -0.3485 | 0.0401 | 0.1571 |
| ENSG00000007866 | -0.3516 | 0.1416 | 0.3655 |
| ENSG00000130522 | -0.3624 | 0.1061 | 0.3034 |
| ENSG00000198816 | -0.3627 | 0.0768 | 0.2458 |
| ENSG00000172493 | -0.3638 | 0.0301 | 0.1282 |
| ENSG00000174306 | -0.3674 | 0.0401 | 0.1571 |
| ENSG00000009307 | -0.3709 | 0.0100 | 0.0574 |
| ENSG00000158055 | -0.3709 | 0.4430 | 0.6790 |
| ENSG00000140044 | -0.3750 | 0.0118 | 0.0652 |
| ENSG00000112658 | -0.3773 | 0.1083 | 0.3071 |
| ENSG00000113916 | -0.3808 | 0.1430 | 0.3678 |
| ENSG00000121690 | -0.3849 | 0.0496 | 0.1823 |
| ENSG00000075426 | -0.3877 | 0.0654 | 0.2207 |
| ENSG00000087903 | -0.3972 | 0.0619 | 0.2126 |
| ENSG00000105856 | -0.4020 | 0.0089 | 0.0530 |
| ENSG00000101493 | -0.4035 | 0.0152 | 0.0784 |
| ENSG00000156650 | -0.4227 | 0.0027 | 0.0215 |
| ENSG00000120068 | -0.4249 | 0.2128 | 0.4598 |
| ENSG00000178163 | -0.4271 | 0.0033 | 0.0253 |
| ENSG00000067646 | -0.4314 | 0.0399 | 0.1565 |
| ENSG00000204789 | -0.4326 | 0.0153 | 0.0789 |
| ENSG00000213762 | -0.4327 | 0.0067 | 0.0427 |
| ENSG00000141905 | -0.4340 | 0.0448 | 0.1697 |
| ENSG00000118526 | -0.4344 | 0.0109 | 0.0613 |
| ENSG00000102935 | -0.4383 | 0.0418 | 0.1617 |
| ENSG00000198807 | -0.4522 | 0.3298 | 0.5681 |
| ENSG00000100968 | -0.4543 | 0.0241 | 0.1095 |
| ENSG00000187079 | -0.4590 | 0.0951 | 0.2822 |
| ENSG00000198753 | -0.4639 | 0.1131 | 0.3159 |
| ENSG00000185650 | -0.4664 | 0.0206 | 0.0978 |
| ENSG00000108799 | -0.4746 | 0.0091 | 0.0537 |
| ENSG00000123358 | -0.4764 | 0.3323 | 0.5700 |
| ENSG00000141579 | -0.4896 | 0.3675 | 0.6055 |
| ENSG00000175395 | -0.5107 | 0.0104 | 0.0592 |
| ENSG00000184271 | -0.5113 | 0.0052 | 0.0353 |
| ENSG00000163659 | -0.5135 | 0.0107 | 0.0603 |
| ENSG00000102038 | -0.5157 | 0.0432 | 0.1655 |
| ENSG00000069011 | -0.5175 | 0.2065 | 0.4517 |
| ENSG00000090661 | -0.5181 | 0.0844 | 0.2609 |
| ENSG00000059728 | -0.5225 | 0.1713 | 0.4044 |
| ENSG00000069667 | -0.5289 | 0.0018 | 0.0160 |
| ENSG00000171223 | -0.5526 | 0.0409 | 0.1593 |
| ENSG00000177508 | -0.5582 | 0.0998 | 0.2916 |
| ENSG00000179388 | -0.5636 | 0.0593 | 0.2063 |
| ENSG00000116044 | -0.5660 | 0.0004 | 0.0046 |
| ENSG00000105996 | -0.5670 | 0.0031 | 0.0237 |
| ENSG00000088854 | -0.5694 | 0.0063 | 0.0408 |
| ENSG00000179277 | -0.5806 | 0.0103 | 0.0586 |
| ENSG00000182568 | -0.5864 | 0.0051 | 0.0353 |
| ENSG00000114853 | -0.5886 | 0.0196 | 0.0944 |
| ENSG00000179981 | -0.5923 | 0.0013 | 0.0120 |
| ENSG00000118495 | -0.6043 | 0.0017 | 0.0153 |
| ENSG00000088881 | -0.6147 | 0.0222 | 0.1034 |
| ENSG00000008441 | -0.6162 | 0.0072 | 0.0451 |
| ENSG00000167081 | -0.6164 | 0.0272 | 0.1194 |
| ENSG00000175745 | -0.6165 | 0.0347 | 0.1415 |
| ENSG00000134138 | -0.6185 | 0.0009 | 0.0089 |
| ENSG00000166173 | -0.6352 | 0.0038 | 0.0279 |
| ENSG00000177606 | -0.6524 | 0.0205 | 0.0975 |
| ENSG00000106004 | -0.6571 | 0.0149 | 0.0774 |
| ENSG00000167800 | -0.6592 | 0.0972 | 0.2862 |
| ENSG00000164736 | -0.6655 | 0.0058 | 0.0384 |
| ENSG00000121297 | -0.6697 | 0.0047 | 0.0331 |
| ENSG00000106571 | -0.6705 | 0.0005 | 0.0062 |
| ENSG00000187098 | -0.6756 | 0.0008 | 0.0087 |
| ENSG00000117318 | -0.6761 | 0.0105 | 0.0595 |
| ENSG00000053254 | -0.6780 | 0.0000 | 0.0003 |
| ENSG00000127528 | -0.6826 | 0.0708 | 0.2330 |
| ENSG00000162599 | -0.6912 | 0.0039 | 0.0284 |
| ENSG00000155090 | -0.6968 | 0.0141 | 0.0741 |
| ENSG00000162772 | -0.6991 | 0.0932 | 0.2784 |
| ENSG00000134874 | -0.7097 | 0.0004 | 0.0048 |
| ENSG00000125968 | -0.7212 | 0.0318 | 0.1329 |
| ENSG00000198846 | -0.7346 | 0.0178 | 0.0880 |
| ENSG00000016082 | -0.7382 | 0.0030 | 0.0232 |
| ENSG00000151623 | -0.7418 | 0.0006 | 0.0069 |
| ENSG00000174282 | -0.7463 | 0.0005 | 0.0055 |
| ENSG00000152784 | -0.7491 | 0.0027 | 0.0214 |
| ENSG00000151090 | -0.7523 | 0.0008 | 0.0087 |
| ENSG00000168874 | -0.7592 | 0.0000 | 0.0001 |
| ENSG00000148516 | -0.7728 | 0.0187 | 0.0910 |
| ENSG00000152284 | -0.8293 | 0.0010 | 0.0100 |
| ENSG00000113580 | -0.8314 | 0.0000 | 0.0000 |
| ENSG00000130844 | -0.8541 | 0.0010 | 0.0096 |
| ENSG00000152217 | -0.8548 | 0.0003 | 0.0034 |
| ENSG00000185630 | -0.8760 | 0.0000 | 0.0008 |
| ENSG00000167074 | -0.8790 | 0.0000 | 0.0005 |
| ENSG00000165821 | -0.8905 | 0.0003 | 0.0041 |
| ENSG00000184828 | -0.9203 | 0.0084 | 0.0509 |
| ENSG00000170345 | -0.9266 | 0.0301 | 0.1281 |
| ENSG00000119950 | -0.9319 | 0.0000 | 0.0001 |
| ENSG00000121413 | -0.9567 | 0.0000 | 0.0003 |
| ENSG00000119508 | -0.9572 | 0.0163 | 0.0824 |
| ENSG00000120693 | -0.9575 | 0.0002 | 0.0023 |
| ENSG00000178573 | -0.9708 | 0.0000 | 0.0006 |
| ENSG00000120738 | -1.0046 | 0.0294 | 0.1261 |
| ENSG00000143995 | -1.0472 | 0.0001 | 0.0010 |
| ENSG00000135218 | -1.1003 | 0.0000 | 0.0009 |
| ENSG00000157514 | -1.1106 | 0.0001 | 0.0013 |
| ENSG00000172201 | -1.1274 | 0.0000 | 0.0000 |
| ENSG00000197576 | -1.1282 | 0.0000 | 0.0009 |
| ENSG00000128016 | -1.1818 | 0.0003 | 0.0039 |
| ENSG00000103241 | -1.2158 | 0.0008 | 0.0083 |
| ENSG00000154556 | -1.2181 | 0.0000 | 0.0002 |
| ENSG00000181449 | -1.2221 | 0.0013 | 0.0118 |
| ENSG00000170577 | -1.3095 | 0.0069 | 0.0438 |
| ENSG00000119138 | -1.3119 | 0.0000 | 0.0003 |
| ENSG00000130700 | -1.3281 | 0.0012 | 0.0117 |
| ENSG00000136826 | -1.3319 | 0.0000 | 0.0000 |
| ENSG00000109906 | -1.3469 | 0.0000 | 0.0002 |
| ENSG00000124440 | -1.3549 | 0.0001 | 0.0022 |
| ENSG00000148826 | -1.3677 | 0.0032 | 0.0247 |
| ENSG00000125740 | -1.3780 | 0.0193 | 0.0933 |
| ENSG00000182938 | -1.3822 | 0.0056 | 0.0374 |
| ENSG00000163328 | -1.3899 | 0.0000 | 0.0000 |
| ENSG00000137273 | -1.4121 | 0.0001 | 0.0011 |
| ENSG00000163884 | -1.4634 | 0.0000 | 0.0000 |
| ENSG00000129194 | -1.5030 | 0.0003 | 0.0042 |
| ENSG00000131668 | -1.7048 | 0.0057 | 0.0379 |
| ENSG00000108924 | -1.7049 | 0.0000 | 0.0000 |
| ENSG00000125285 | -1.8892 | 0.0001 | 0.0012 |
| ENSG00000164107 | -2.0375 | 0.0003 | 0.0034 |
